# Diaportones A–C: Three New Metabolites From Endophytic Fungus *Diaporthe foeniculina* BZM-15

**DOI:** 10.3389/fchem.2021.755351

**Published:** 2021-11-19

**Authors:** Fenghua Kang, Xiuxiang Lu, Sha Zhang, Dekun Chen, Min Kuang, Weiwei Peng, Jianbing Tan, Kangping Xu, Zhenxing Zou, Haibo Tan

**Affiliations:** ^1^ Xiangya School of Pharmaceutical Sciences, Central South University, Changsha, China; ^2^ Hunan Key Laboratory of Diagnostic and Therapeutic Drug Research for Chronic Diseases, Central South University, Changsha, China; ^3^ South China Botanical Garden, Chinese Academy of Sciences, Guangzhou, China

**Keywords:** endophytic fungus, *Diaporthe foeniculina*, *Leptospermum brachyandrum*, diaportone, structure elucidation, cytotoxicity

## Abstract

Phytochemical investigation of *Diaporthe foeniculina* BZM-15 led to one new *γ*-butyrolactone derivative, diaportone A (**1**), one cyclopentenone derivative, diaportone B (**3**), and one monoterpene derivative, diaportone C (**5**), along with six known compounds (**2**, **4**, and **6–9**). Their structures as well as the absolute configurations were characterized by means of NMR, HRESIMS, and ECD spectroscopy and quantum chemistry calculation, respectively. Furthermore, all compounds were evaluated for their cytotoxic activity and antibacterial activity, and compounds **7** and **8** displayed significant antiproliferative effects on three human cancer cell lines (SF-268, MCF-7, and HepG2) with IC_50_ values ranging from 3.6 to 15.8 μM.

## Introduction

**FIGURE 1:**
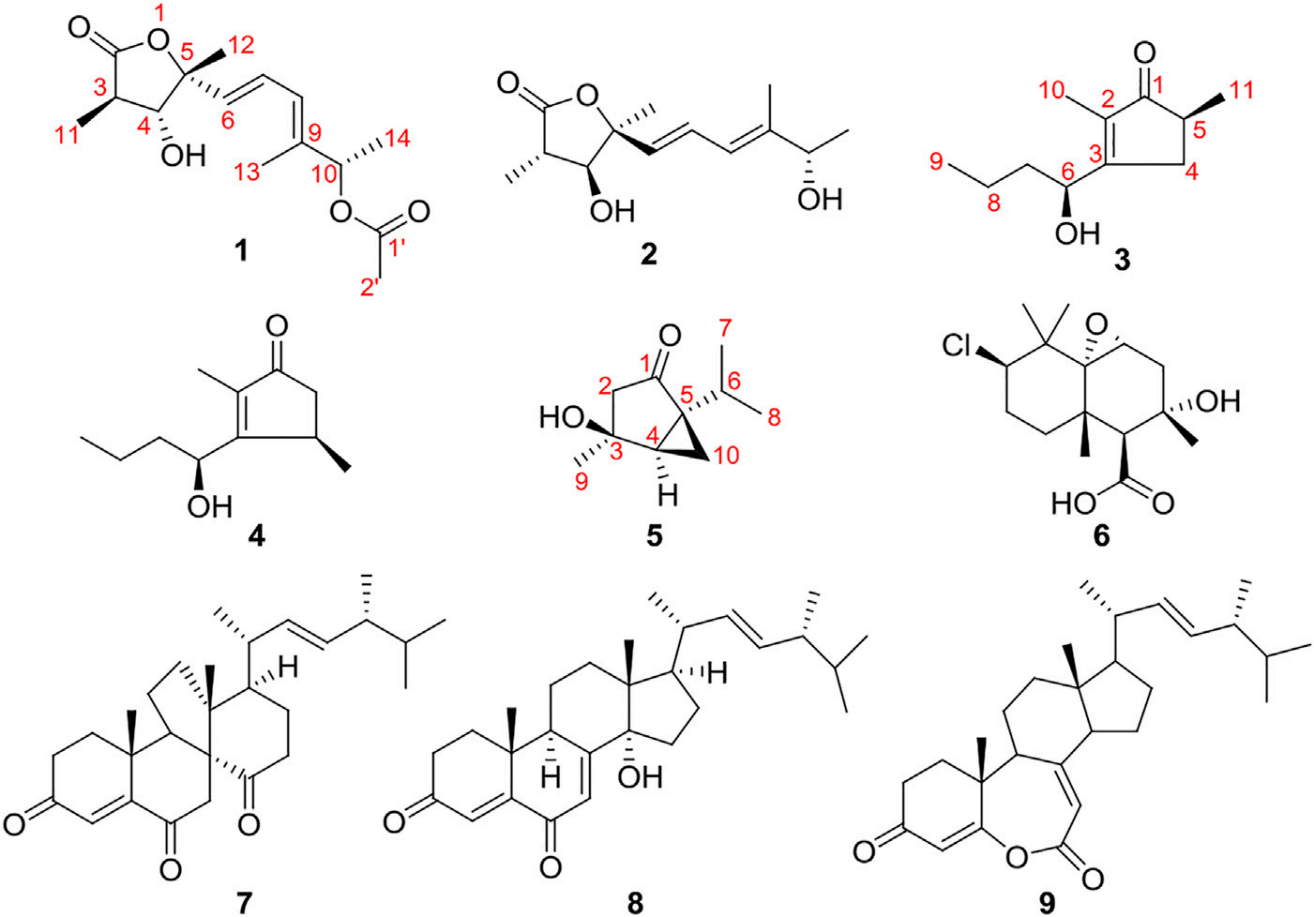
Chemical structures of compounds **1–9**.

Fungi are prolific producers of bioactive secondary metabolites and have contributed to improvements in human and animal health in spectacular and indispensable ways (Bills and Gloer, 2016; El-Elimat et al., 2021). They are ubiquitous in nature and often provide nutrients or protection for the host (Wani et al., 2015). Many endophytic fungi produce compounds with a novel structure and specific bioactivity for their long period of living in host tissues, which have been potential resources for novel compounds and new drugs (Jia et al., 2016; Kaul et al., 2012). *Leptospermum brachyandrum* is a famous ornamental and medicinal plant that belongs to the *Myrtaceae* family. In our previous research, three endophytes were isolated from this plant, such as *Diaporthe foeniculina*, *Eutypella scoparia*, and *Rhytidhysteron* sp., and a number of novel natural products with antibacterial or cytotoxic activity have been discovered from these endophytes (Zhang et al., 2021a; Zhang et al., 2021b; Zhang et al., 2020; Zhang et al., 2021). Simultaneously, five new 2-pyrones were isolated from *D. foeniculina* (Yu et al., 2021). With the aim of seeking new bioactive natural products from medicinal plant endophytes, chemical investigation of strain *D. foeniculina* BZM-15 was further researched and afforded to find three new metabolites, including one *γ*-butyrolactone derivative, diaportone A (**1**), one cyclopentenone derivative, diaportone B (**3**), and one monoterpene, diaportone C (**5**), along with six known compounds, colletolides A (**2**) (Li et al., 2019), phomotenone (**4**) (Ahmed et al., 2011), altiloxin B (**6**) (Hemberger et al., 2013), dankasterone A (**7**) (Jiao et al., 2015), 14*α*-hydroxyergosta-4,7,22-triene-3,6-dione (**8**) (Liu et al., 2018), and fortisterol (**9**) (Kitchawalitet al., 2014) (Figure 1). Herein, this report describes the isolation, structural elucidation, and biological activity of compounds **1–9**.

## Materials and Methods

### General Experimental Procedures

Optical rotations were measured using an Anton Paar MCP-500 spectropolarimeter (Anton Paar, Graz, Austria). The NMR spectra were recorded on a Bruker Avance-500 spectrometer (Bruker Corporation, Fällanden, Switzerland) with TMS as an internal reference. Experimental ECD spectra in MeOH were acquired in a quartz cuvette of 1 mm optical path length on an Applied Photophysics Chirascan spectrometer. HRESIMS spectra were obtained in a Thermo MAT95XP high-resolution mass spectrometer (Thermo Fisher Scientific, Bremen, Germany). Preparative HPLC was performed on an Agilent 1260 Infinity system equipped with a DAD detector using a preparative YMC ODS C_18_ column (20 × 250 mm, 5 μm). Column chromatography was performed using silica gel (200–300 mesh, Qingdao Marine Chemical Inc., Qingdao, China) and Sephadex LH-20 gel (Pharmacia Fine Chemical Co. Ltd., Sweden). Thin-layer chromatography (TLC) was carried out on silica gel plates (Merck KGaA, Darmstadt, Germany) using various solvent systems. All solvents were purchased from Guangzhou Chemical Reagent Company, Ltd. (Guangzhou, China).

### Cultivation and Culture Extraction

The fungus *D. foeniculina* BZM-15 was isolated from the plant *Leptospermum brachyandrum*, which was collected from South China Botanical Garden (SCBG), Chinese Academy of Sciences, China, in September 2016. The strain was identified as *D. foeniculina* according to the sequence analysis of rDNA ITS (internal transcribed spacer) region, which has been submitted to GenBank with the accession number of MN788609. The strain was deposited in the Laboratory of Natural Product Medicinal Chemistry, SCBG.

The fungus *D. foeniculina* BZM-15 was incubated in 200 ml of potato dextrose broth at 30°C on a rotary shaker (120 rpm) for 7 days to acquire the seed broth. Large-scale fermentation was carried out in Erlenmeyer flasks (16 × 3 L); each contained rice (200 g) and distilled water (300 ml), which were autoclaved at 121 °C for 25 min. After cooling at room temperature, seed broth was added to those Erlenmeyer flasks, which were fermented for 30 days at 28°C. After cultivation, the obtained mycelial solid medium was extracted with EtOAc (three times, 24 h for every time) at room temperature, and the extract solution was concentrated *in vacuo* to receive a crude extract (50 g).

### Isolation of Compounds 1–9

The crude extract was fractionated by silica gel column chromatography ((CC) (PE-EtOAc v/v, 100:1-0:100)) to afford six main fractions (Fr.1–Fr.6). Fr.5 (7.22 g) was divided into ODS CC and eluted with MeOH-H_2_O (v/v, 40–100%) to give six subfractions (Fr.5-1 to Fr.5-6). Fr.5-2 (1.94 g) was separated by Sephadex LH-20 CC, eluting with CHCl_3_-MeOH (v/v, 1:1) to provide five subfractions (Fr.5-2-1 to Fr.5-2-5). Fr.5-2-2 (1.23 g) was chromatographed using CC on silica gel, eluted with CHCl_3_-MeOH (v/v, 100:0-0:100), and then further purified by semipreparative HPLC with CH_3_CN-H_2_O (40: 60) to give compound **5** (4.0 mg). Compound **6** (5.3 mg) was obtained from Fr.5-2-3 on a semipreparative HPLC with CH_3_CN-H_2_O (35:65). Fr.5-3 (405.0 mg) was separated by Sephadex LH-20 CC, eluting with CHCl_3_-MeOH (v/v, 1:1) to provide five subfractions (Fr.5-3-1 to Fr.5-3-5). Fr.5-3-1 (211.4 mg) was isolated by column chromatography on silica gel and eluted with CHCl_3_-MeOH (v/v, 10:1-0:1) to get three fractions (Fr.5-3-1-1 to Fr.5-3-1-3). Fr.5-3-1-2 (11.4 mg) was then subjected to semipreparative HPLC with CH_3_CN-H_2_O (v/v, 50:50) to afford compounds **1** (8.0 mg) and **2** (6.1 mg). Fr.5-3-2 (80.3 mg) was separated by semipreparative HPLC with CH_3_CN-H_2_O (v/v, 50:50) to afford compounds **3** (3.9 mg) and **4** (4.3 mg).

Fr.3 (3.2 g) was subjected to CC on silica gel eluted with a gradient system of PE-EtOAc (v/v, 20:0–0:100) to afford five fractions (Fr.3-1 to Fr.3-5). Fr.3-2 (890 mg) was separated on a Sephadex LH-20 column with MeOH and then separated by preparative HPLC using CH_3_CN-H_2_O (80:20) to provide compounds **7** (5.8 mg) and **9** (4.4 mg). Fr.3-3 (635 mg) was purified by silica gel CC and eluted with a gradient system of CH_2_Cl_2_-MeOH (v/v, 100:0–0:100) to provide compound **8** (10.3 mg).

*Diaportone A (**1**)*: colorless oil; ${\left[\alpha \right]}_{D}^{25}$ – 3.7 (*c* 1.0, MeOH); UV (MeOH): *λ*_max_ (log *ε*): 238 (3.27) nm; IR *ν*_max_: 3,325 and 1,635, 667 cm^−1^; HRESIMS: *m/z* 305.1361 [M + Na] ^+^ (calcd for C_15_H_22_NaO_5_, 305.1359). ^1^H (500 MHz); and ^13^C (125 MHz) NMR data, see Table 1.

**TABLE 1:** ^1^H (500 MHz) and ^13^C (125 MHz) NMR spectral data of compound **1** in CD_3_OD.

No	*δ*_H_ (*J* in Hz)	*δ*_C_, type	No	*δ*_H_ (*J* in Hz)	*δ*_C_, type
2	—	178.5, C	10	5.25, q (7.0)	76.4, CH
3	2.48, s	42.6, CH	11	1.22, d (7.0)	12.4, CH_3_
4	3.80, d (10.5)	82.5, CH	12	1.51, s	25.2, CH_3_
5	—	86.6, C	13	1.76, s	12.7, CH_3_
6	5.92, d (15.5)	133.3, CH	14	1.34, d (6.5)	19.4, CH_3_
7	6.48, dd (15.5, 10.5)	126.4, CH	1′	—	172.2, C
8	6.10, d (10.5)	126.1, CH	2′	2.03, s	21.1, CH_3_
9	—	139.2, C	—	—	—

*Diaportone B (**3**)*: yellow oil; ${\left[\alpha \right]}_{D}^{25}$ + 14.5 (*c* 0.8, MeOH); UV (MeOH): *λ*_max_ (log *ε*): 236 (3.07) nm; IR *ν*_max_: 3,415, 2,960, 2,931, 2,872, 1,681, 1,639, 1,456, 1,381, 1,336, 1,172, and 1,026 cm^−1^; HRESIMS: *m/z* 205.1206 [M + Na] ^+^ (calcd for C_11_H_18_NaO_2_, 205.1199). ^1^H (500 MHz); and ^13^C (125 MHz) NMR data, see Table 2.

**TABLE 2:** ^1^H (500 MHz) and ^13^C (125 MHz) NMR spectral data of compounds **3** and **5** in acetone-*d*_6_.

No	3		5	
	*δ*_H_ (*J* in Hz)	*δ*_C_, type	*δ*_H_ (*J* in Hz)	*δ*_C_, type
1	—	212.2, C	—	210.6, C
2	—	134.4, C	1.88, d (17.0)	49.1, CH_2_
			2.36, m	
3	—	173.7, C	—	71.7, C
4	2.08, m	34.8, CH_2_	1.97, dd (7.5, 4.0)	38.4, CH
	2.98, m			
5	2.25, m	39.9, CH	—	46.1, C
6	4.75, dd (8.0, 5.5)	69.5, CH	1.88, m	27.2, CH
7	1.53, m	38.7 CH_2_	0.95, d (7.0)	20.0, CH_3_
	1.69, m			
8	1.35, m	19.6, CH_2_	0.91, d (7.0)	19.6, CH_3_
	1.45, m			
9	0.93, t (7.0)	14.6, CH_3_	1.37, s	29.9, CH_3_
10	1.65, s	8.7, CH_3_	1.10, ddd (7.5, 5.0, 1.5)	16.6, CH_2_
			1.36, dd (5.0, 4.5)	
11	1.08, d (7.5)	17.2, CH_3_	—	—

*Diaportone C (**5**)*: white solid; ${\left[\alpha \right]}_{D}^{25}$ + 10.3 (*c* 0.5, MeOH); UV (MeOH): *λ*_max_ (log *ε*): 200 (2.59) nm, 273 (1.39) nm; IR *ν*_max_: 3,431, 1962, 2,873, 1708, 1,458, 1,375, 1,259, 1,182, 1,130, 1032,999,945, 758, and 682 cm^−1^; HRESIMS: *m/z* 191.1051 [M + Na] ^+^ (calcd for C_10_H_16_NaO_2_, 191.1043). ^1^H (500 MHz); and ^13^C (125 MHz) NMR data, see Table 2.

### ECD Calculation Methods

The ECD spectra of compounds **1**, **3**, and **5** were calculated by using the Gaussian09 package (Frisch et al., 2016). Each of their configuration was optimized at the B3LYP-D3(BJ)/TZVP (IEFPCM) level of theory. The theoretic ECD spectra were calculated on the mPW1PW91/6-311G* (IEFPCM) level of theory and Boltzmann average was calculated for the spectra according to Gibbs free energy. SpecDis v1.71 was used to simulate an ECD curve with a sigma/gamma value of 0.3 eV^2^ (Bruhn et al., 2013). The calculated ECD curves of compounds **1** and **3** were red shifted and blue shifted by 5 nm, respectively.

### Antibacterial Assay

Antibacterial activity of all compounds against MRSA (JCSC 3063) and *E. coli* (ATCC 8739) was tested by the broth macrodilution method on 96-well plates according to the CLSI recommendation (Li et al., 2014). Vancomycin (MIC = 1.25 µg/ml) was used as a positive control.

### Cytotoxicity Assay

The cytotoxicity of all compounds against three human tumor cell lines, SF-268 (CNS cancer), MCF-7 (breast cancer), and HepG2 (hepatoma cancer), and normal cell line LX-2 was tested using the MTT assay. Adriamycin was used as a positive control. All cells were seeded into 96-well plates at 5 × 10^4^ cells/ml and incubated at 37°C under a 5% CO_2_ atmosphere for 24 h. Then, the tested compounds were added. After 72 h, MTT solution was added into each well, which was further incubated. The cell-free supernatant was removed and formazan crystals were subsequently dissolved in DMSO. Optical density (OD) was recorded at 490 nm on a microplate reader to calculate the IC_50_ values.

## Results and Discussion

Compound **1** was isolated as colorless oil and assigned the molecular formula C_15_H_22_O_5_ as inferred from its HRESIMS ion peak at *m/z* 305.1361 [M + Na] ^+^ (calcd 305.1359 for C_15_H_22_NaO_5_). The ^1^H NMR spectral data (Table 1), in combination with the HSQC spectrum, displayed two ester carbonyl groups [*δ*_C_ 178.5 (C-2) and 172.2 (C-1′)], one conjugated diene [*δ*_H_ 5.92 (H-6), 6.48 (H-7), and 6.10 (H-8); *δ*_C_ 133.3 (C-6), 126.4 (C-7), 126.1 (C-8), and 139.2 (C-9)], two oxygenated methines [*δ*_H_ 5.25 (H-10) and 3.80 (H-4); *δ*_C_ 76.4 (C-10) and 82.5 (C-4)], one methine [*δ*_H_ 2.48 (H-3); *δ*_C_ 42.6 (C-3)], one oxygen quaternary carbon [*δ*_C_ 86.6 (C-5)], and five methyl groups [*δ*_H_ 1.22 (H-11), 1.51 (H-12), 1.76 (H-13), 1.31 (H-14), and *δ*_H_ 2.03 (H-2′); *δ*_C_ 12.4 (C-11), 25.2 (C-12), 12.7 (C-13), 19.4 (C-14), and 21.1 (C-2′)].

Compared to the NMR spectra of compound **2** (Table 1), they shared a typical *γ*-butyrolactone ring bearing two methyl groups at C-3 and C-5 and a hydroxyl group at C-4. However, the spectra of compound **1** exhibited additional signals for the acetyl group, which was connected with 10-OH to form an ester. This conclusion can be proved by the correlations of H-10 with C-1′ in the HMBC spectrum. Thus, the planar structure of compound **1** was elucidated, as shown in Figure 1.

**FIGURE 2:**
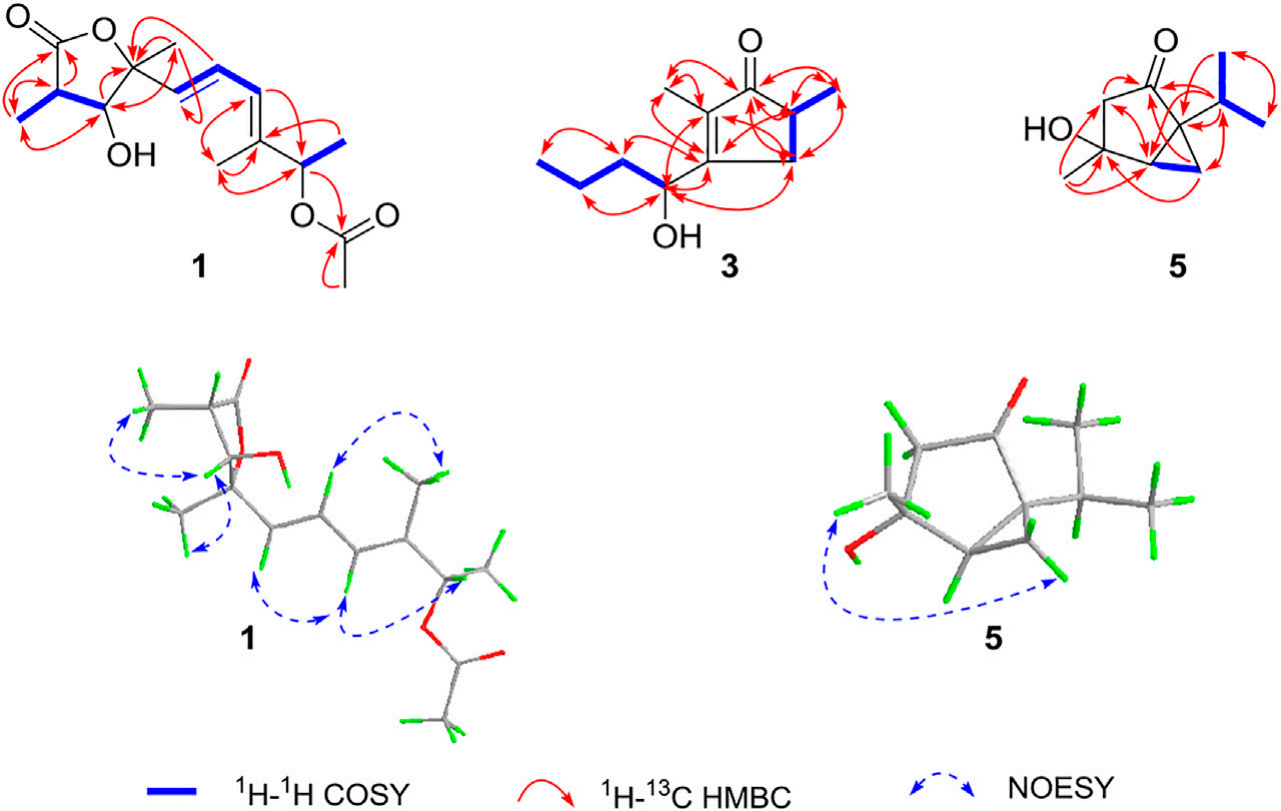
^1^H-^1^H COSY, Key HMBC, and NOESY correlations of compounds **1**, **3**, and **5**.

**FIGURE 3:**
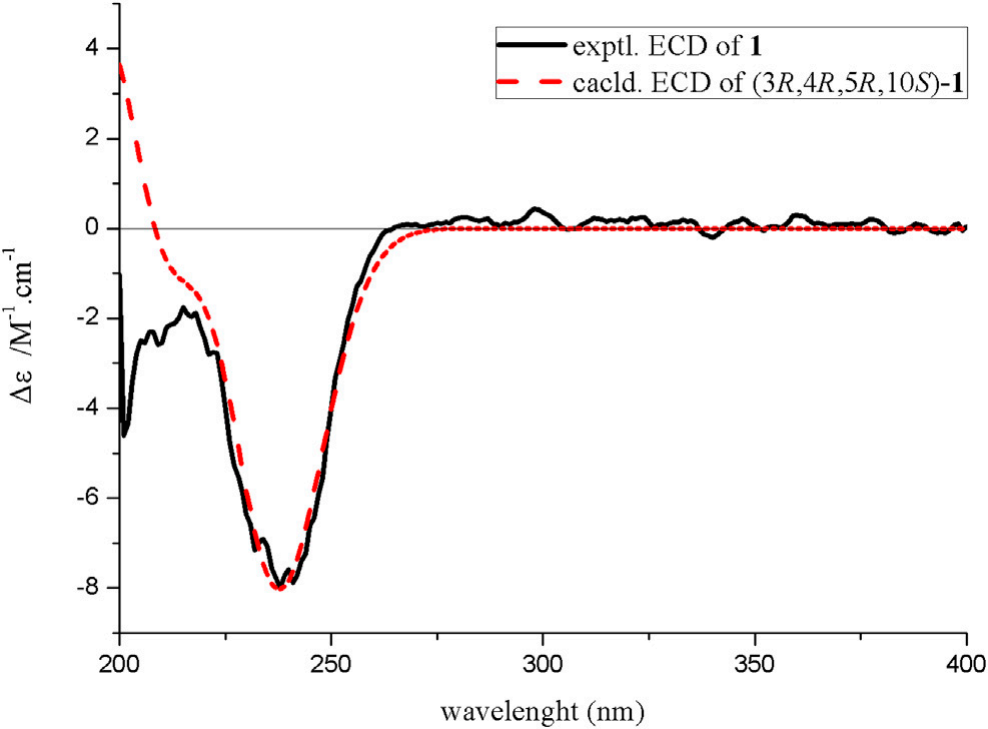
Experimental and calculated ECD spectra of compound **1**.

The relative configuration was determined by NOESY spectrum and coupling constants. The NOE correlations of H-11/H-4/H-12 indicated that 11-H_3_, 4-H, and 12-H_3_ were on the same side of the lactone ring. A conjugated diene moiety was determined by the large coupling constant *J* = 15.5 Hz between H-6 and H-7 that was *trans*-oriented and *J* = 10.5 Hz between H-7 and H-8 that was *cis*-oriented. In addition, the *E* confirmation of 8,9-diene was deduced from the NOE cross peaks of H-6/H-8/H-10 and H_3_-13/H-7 (Figure 2). The chiral HPLC analysis of compound **1** revealed that it should be optically pure. The calculated ECD spectrum was consistent with its experimental ECD spectrum, suggesting the absolute configuration of compound **1** as 3*R*,4*R*,5*R*,10*S* (Figure 3). Thus, the structure of compound **1** was determined as a new *γ*-butyrolactone derivative with 6,8-hexadien-1-ol,1-acetate side chain and was named diaportone A. Notably, diaportone A possess conjugated double bond, which might be light sensitive. Moreover, it might be an artificial compound generated from the known compound **2** through acylation, although much more evidence was needed.

**FIGURE 4:**
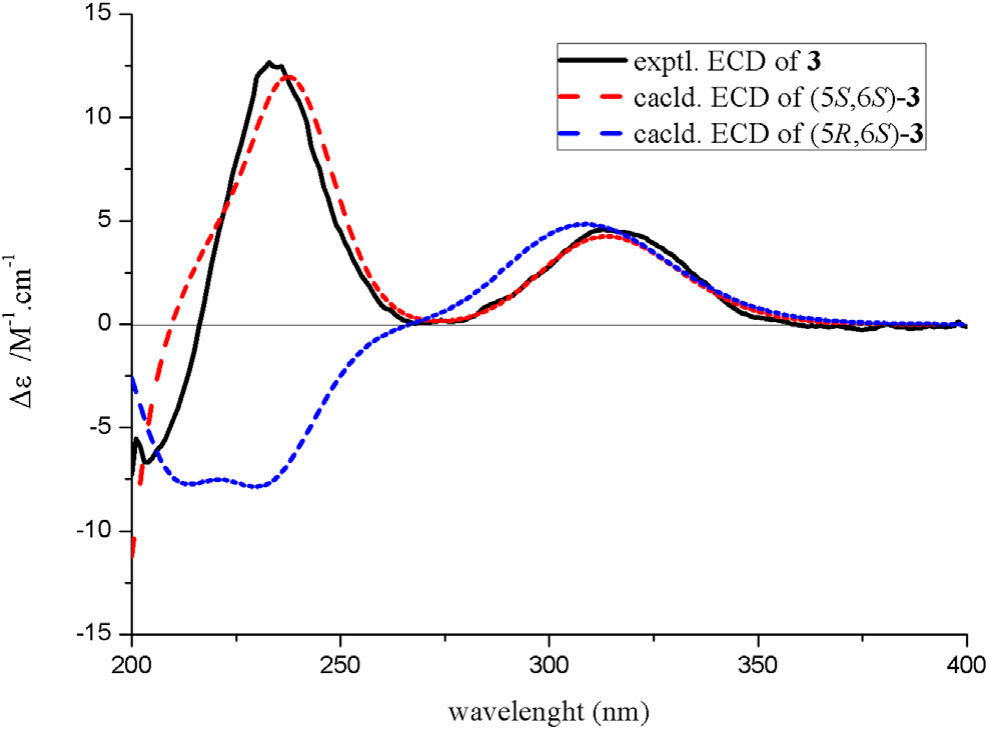
Experimental and calculated ECD spectra of compound **3**.

Compound **3** was isolated as yellow oil. It was determined to have a molecular formula of C_11_H_18_O_2_ by a combination of its HRESIMS ion peak at *m/z* 183.1388 [M + H] ^+^ (calcd 182.1307 for C_11_H_19_O_2_). The ^1^H and ^13^C NMR spectroscopic data (Table 2) of compound **3** exhibited characteristic signals assignable to a conjugated ketone carbonyl, a tetrasubstituted olefin, two methines (one oxygenated), three methylene groups, and three methyl groups. A detailed analysis of the NMR data exhibited that compound **3** was similar to the known compound **4** (Ahmed et al., 2011) (Table 2) with the only difference in compound **3** being the substitution position of CH_3_-11, which was deduced based on the HMBC cross peaks of H_3_-11 (*δ*_H_ 1.08)/C-1 (*δ*_C_ 212.2), C-5 (*δ*_C_ 39.9), and C-4 (*δ*_C_ 34.8) (Figure 2). The absolute configuration of compound **3** was confirmed by the similarity between the calculated ECD curve of 5*S*,6*S* and its experimental ECD spectrum (Figure 4). Therefore, the structure of compound **3**, diaportone B, was defined as shown.

Compound **5** was isolated as a white solid. The molecular formula C_10_H_16_O_2_ was determined by the HRESIMS ion peak at *m/z* 169.1224 [M + H] ^+^ (calcd 169.1229 for C_10_H_17_O_2_). The 1D NMR data (Table 2) of compound **5** exhibited three methyl groups, two methylene groups, two methines, two sp^3^ nonprotonated carbons (one oxygenated), and one ketone carbonyl. Comparison of NMR data with those of dihydroxysabinane (Lin et al., 2009) revealed a high degree of similarity skeleton, where the only obvious difference is in the presence of a carbonyl group at C-1 in compound **5** instead of a hydroxyl group in dihydroxysabinane. This deduction was confirmed by the HMBC correlations of C-1 with H-2 (*δ*_H_ 1.88 and 2.36), H-6 (*δ*_H_ 1.88), and H-10 (*δ*_H_ 1.36 and 1.10) and obvious low-field chemical shift of C-1 (*δ*_C_ 210.9) (Figure 2).

**FIGURE 5:**
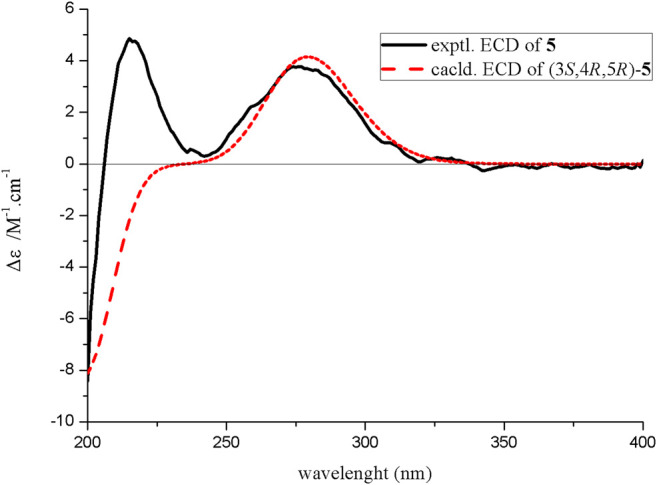
Experimental and calculated ECD spectra of compound **5**.

The relative configuration of compound **5** was determined by the analysis of ROESY spectral data (Figure 2). The methylene (CH_2_-10) and the hydroxyl groups at the chiral carbons (C-3) were assigned as *β*-oriented and the methyl groups (CH_3_-9) and the methine (CH-6) should be *α*-oriented due to the presence of the ROESY correlation of H-4 with H_3_-9. The above data supported the presence of two possible enantiomers (3*S*,4*R*,5*R*)-**5** and (3*R*,4*S*,5*S*)-**5**. To determine the absolute configuration of compound **5**, the ECD calculation was performed. Its experimental ECD spectrum of compound **5** was in good agreement with the calculated ECD spectrum for 3*S*,4*R*,5*R* (Figure 5). Therefore, the structure of compound **5** was determined and given the trivial name diaportone C.

Finally, the cytotoxic activities of all compounds inhibited three human cancer cell lines (SF-268, MCF-7, and HepG2), and antibacterial activities against MRSA and *E. coli* were evaluated. As show in Table 3, compounds **7** and **8** showed different potency of cytotoxicity against the three cell lines, with IC_50_ values ranging from 3.6 to 15.8 μM, whereas they were not active on the normal cell line LX-2. Unfortunately, none of the compounds showed any antibacterial activities against MRSA and *E. coli* at a concentration of 100 µg/ml.

**TABLE 3:** Cytotoxicity of compounds **1–9** against three human cancer cell lines.

Compounds	IC_50_ ^a^ (μM)				SI Values^b^
	SF268	MCF-7	HepG2	LX-2	
**1–6**, **9**	>40	>40	>40	>40	NA
**7**	6.7 ± 0.7	3.6 ± 1.1	4.9 ± 0.9	>40	>6
**8**	8.3 ± 0.5	11.4 ± 0.3	15.8 ± 1.3	>40	>5
Adriamycin^c^	1.9 ± 0.03	1.5 ± 0.01	2.2 ± 0.03	>40	>21

a Results are the mean ± SD (*n* = 3).

b Safety index (SI) value = IC_50_ for LX-2 cell line/IC_50_ for SF286 cell line.

c Positive control.

NA, not active.

## Conclusion

A phytochemical investigation into *D. foeniculina* BZM-15 resulted in the isolation and structural elucidation of three undescribed and six known compounds. Their structures including absolute configurations were determined by extensive physicochemical and spectroscopic analysis, as well as by ECD calculation. Cytotoxicity assays found that compounds **7** and **8** showed good cell inhibition against three human cancer cell lines (SF-268, MCF-7, and HepG2). This result enriched the study on the chemical constituents of *D. foeniculina* and validated that endophytic fungi remained a rich source of structurally/biologically new compounds.

## Data Availability

The original contributions presented in the study are included in the article/Supplementary Material; further inquiries can be directed to the corresponding authors.
