# Supplementary material for: Diaportones A–C: Three New Metabolites From Endophytic Fungus Diaporthe foeniculina BZM-15
Source: Front Chem. 2021 Nov 19;9:755351. doi: 10.3389/fchem.2021.755351 (PMC8640094; doi:10.3389/fchem.2021.755351)
Supplement: Supplementary file 2 [file DataSheet1.docx]

Supplementary Material

Diaportones A-C, Three New Metabolites from Endophytic Fungus Diaporthe Foeniculina BZM-15

Fenghua Kang^1,2,†^, Xiuxiang Lu^3,†^, Sha Zhang^1,2^, Dekun Chen^1,2^, Min Kuang^1,2^, Weiwei Peng^1,2^, Jianbing Tan^1,2^, Kangping Xu^1,2^, Zhenxing Zou^1,2*^, Haibo Tan^1,2,3*^

^1^Xiangya School of Pharmaceutical Sciences, Central South University, Changsha, China

^2^Hunan Key laboratory of Diagnostic and Therapeutic Drug Research for Chronic Diseases, Central South University, Changsha, China

^3^South China Botanical Garden, Chinese Academy of Sciences, Guangzhou, China

**^†^**These authors contributed equally to this work.

*** Correspondence:**Zhenxing Zou
[zouzhenxing@csu.edu.cn](mailto:zouzhenxing@csu.edu.cn)

Haibo Tan

tanhaibo@scbg.ac.cn

# Content Page

**Diaportone A (1)**

**Supplementary Figure 1**. HRESIMS spectrum of **1** 3

**Supplementary Figure 2.** IR spectrum of **1** 3

**Supplementary Figure 3**. UV spectrum of **1** 4

**Supplementary Figure 4**. ^1^H NMR spectrum (500 MHz) of **1** in CD_3_OD 4

**Supplementary Figure 5**. ^13^C NMR spectrum (125 MHz) of **1** in CD_3_OD 5

**Supplementary Figure 6**. ^1^H-^1^H COSY spectrum of **1** in CD_3_OD 5

**Supplementary Figure 7**. HSQC spectrum of **1** in CD_3_OD 6

**Supplementary Figure 8**. HMBC spectrum of **1** in CD_3_OD 6

**Supplementary Figure 9**. NOESY spectrum of **1** in CD_3_OD 7

**Diaportone B (3)**

**Supplementary Figure 10**. HRESIMS spectrum of **3** 7

**Supplementary Figure 11.** IR spectrum of **3** 8

**Supplementary Figure 12**. UV spectrum of **3** 8

**Supplementary Figure 13**. ^1^H NMR spectrum (500 MHz) of **3** in acetone-*d*_6_ 9

**Supplementary Figure 14**. ^13^C NMR spectrum (125 MHz) of **3** in acetone-*d*_6_ 9

**Supplementary Figure 15**. ^1^H-^1^H COSY spectrum of **3** in acetone-*d*_6_ 10

**Supplementary Figure 16**. HSQC spectrum of **3** in acetone-*d*_6_ 10

**Supplementary Figure 17**. HMBC spectrum of **3** in acetone-*d*_6_ 11

**Supplementary Figure 18**. NOESY spectrum of **3** in acetone-*d*_6_ 11

**Diaportone C (5)**

**Supplementary Figure 19**. HRESIMS spectrum of **5** 12

**Supplementary Figure 20.** IR spectrum of **5** 12

**Supplementary Figure 21**. UV spectrum of **5** 13

**Supplementary Figure 22**. ^1^H NMR spectrum (500 MHz) of **5** in acetone-*d*_6_ 13

**Supplementary Figure 23**. ^13^C NMR spectrum (125 MHz) of **5** in acetone-*d*_6_ 14

**Supplementary Figure 24**. ^1^H-^1^H COSY spectrum of **5** in acetone-*d*_6_ 14

**Supplementary Figure 25**. HSQC spectrum of **5** in acetone-*d*_6_ 15

**Supplementary Figure 26**. HMBC spectrum of **5** in acetone-*d*_6_ 15

**Supplementary Figure 27**. NOESY spectrum of **5** in acetone-*d*_6_ 16

**Supplementary Figure 28**. Dose-response curves for three human cancer cell lines (SF-268, MCF-7, and HepG2) and a normal cell line (LX-2) exposed to compounds **1-9**. 16

**Supplementary Data 1.** Cartesian Coordinates of stable conformers for diaportone A (**1**) 17

**Supplementary Data 2.** Cartesian Coordinates of stable conformers for diaportone B (**2**) 33

**Supplementary Data 2.** Cartesian Coordinates of stable conformers for diaportone C (**5**) 42


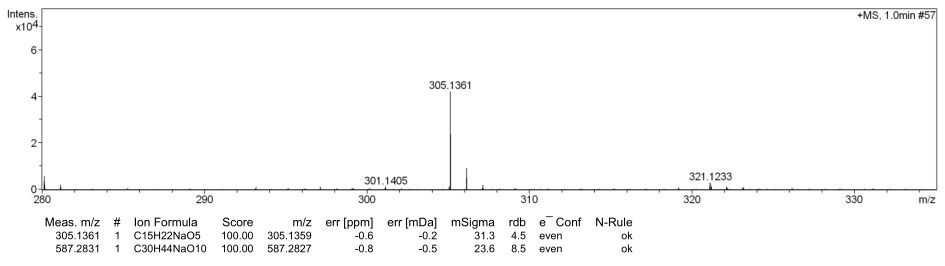


**Supplementary Figure 1**. HRESIMS spectrum of **1**

**Supplementary Figure 2.** IR spectrum of **1**.

**

**

**Supplementary Figure 3**. UV spectrum of **1**

**
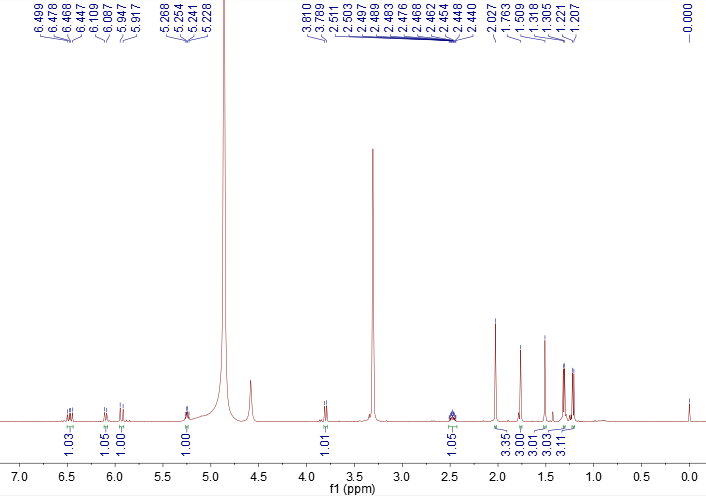
**

**Supplementary Figure 4**. ^1^H NMR spectrum (500 MHz) of **1** in CD_3_OD

**
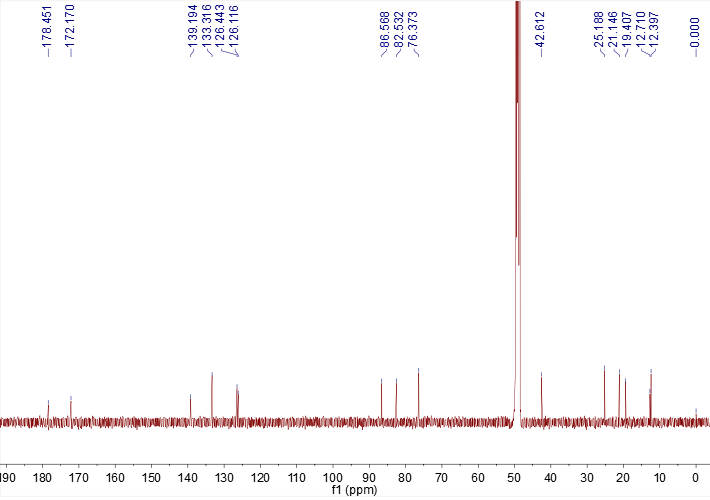
**

**Supplementary Figure 5**. ^13^C NMR spectrum (125 MHz) of **1** in CD_3_OD

**
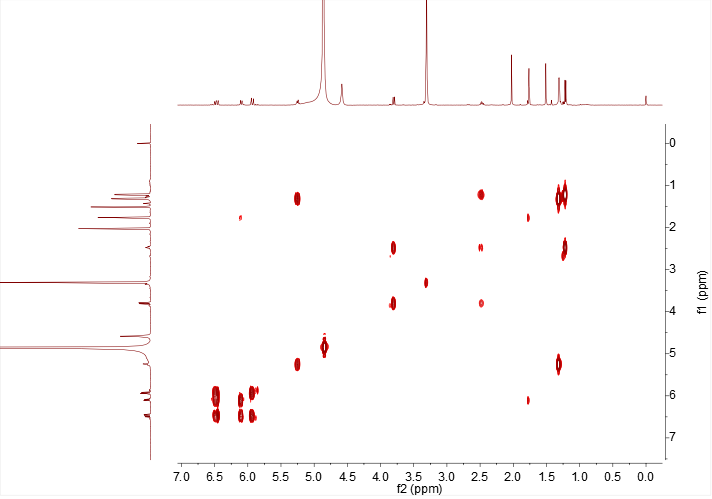
**

**Supplementary Figure 6**. ^1^H-^1^H COSY spectrum of **1** in CD_3_OD


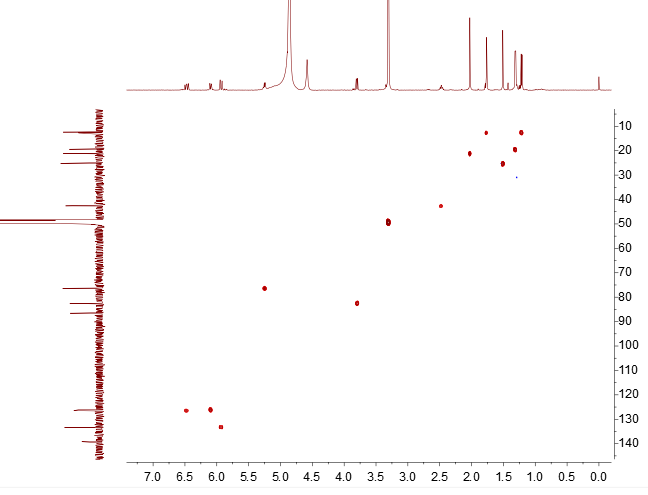


**Supplementary Figure 7**. HSQC spectrum of **1** in CD_3_OD


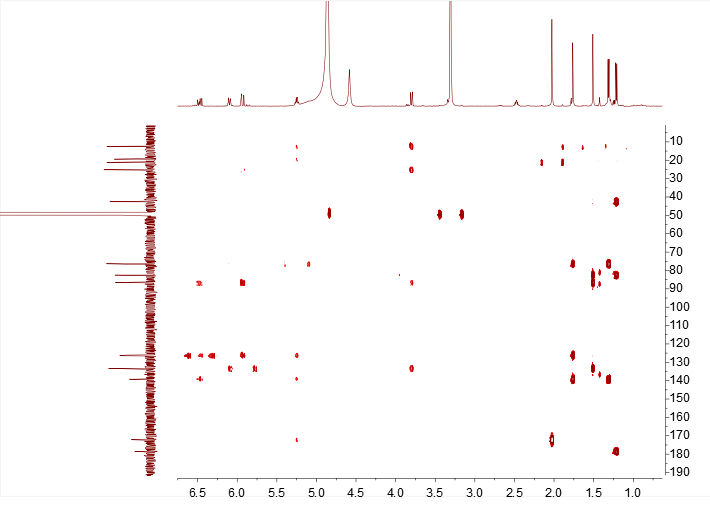


**Supplementary Figure 8**. HMBC spectrum of **1** in CD_3_OD

**
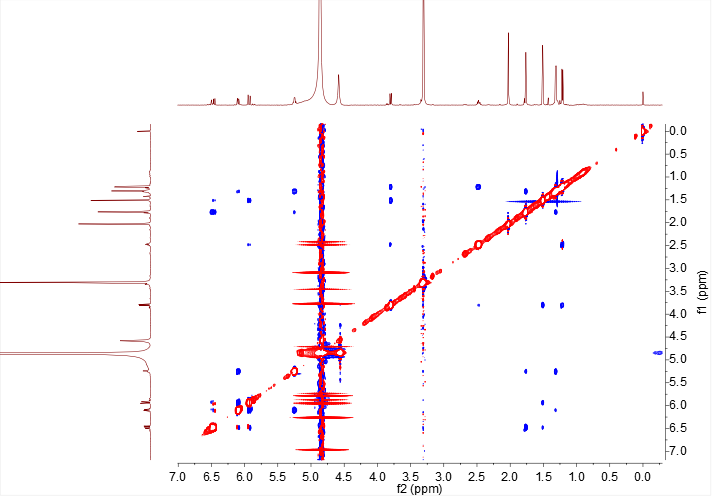
**

**Supplementary Figure 9**. NOESY spectrum of **1** in CD_3_OD

**
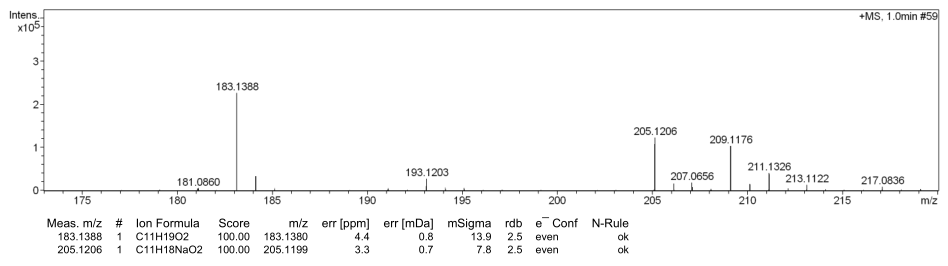
**

**Supplementary Figure 10**. HRESIMS spectrum of **3**

**Supplementary Figure 11.** IR spectrum of **3**

**

**

**Supplementary Figure 12**. UV spectrum of **3**

**
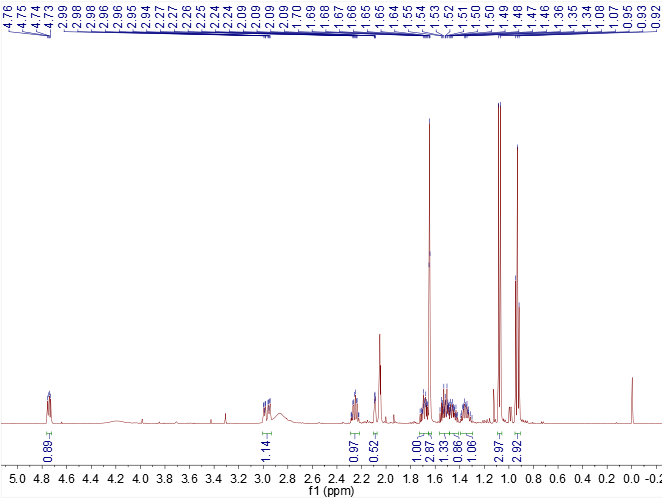
**

**Supplementary Figure 13**. ^1^H NMR spectrum (500 MHz) of **3** in acetone-*d*_6_

**
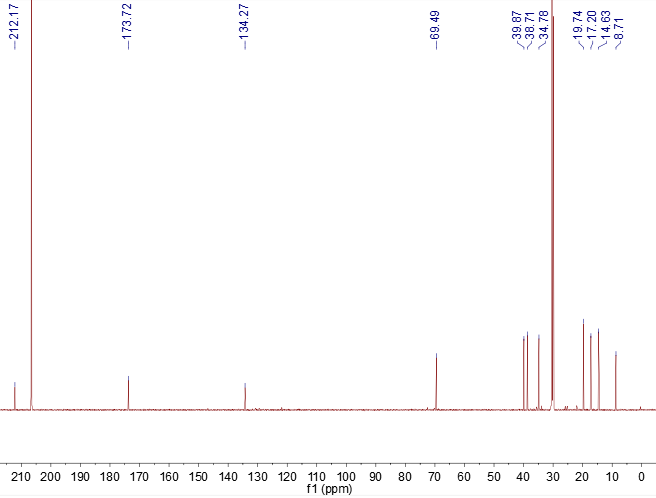
**

**Supplementary Figure 14**. ^13^C NMR spectrum (125 MHz) of **3** in acetone-*d*_6_

**
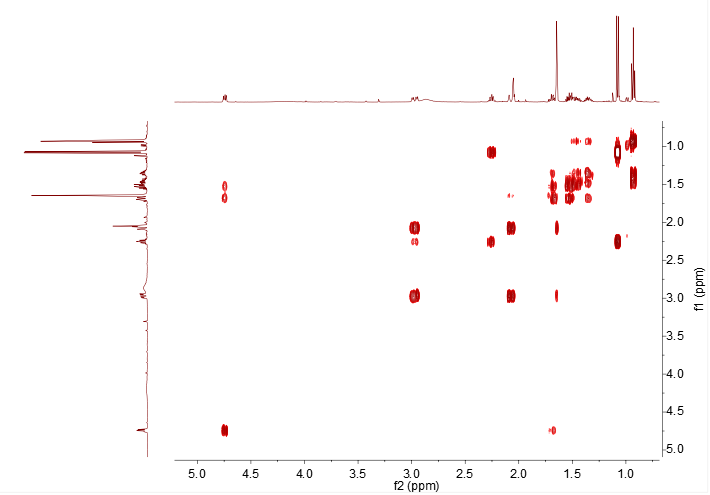
**

**Supplementary Figure 15**. ^1^H-^1^H COSY spectrum of **3** in acetone-*d*_6_

**
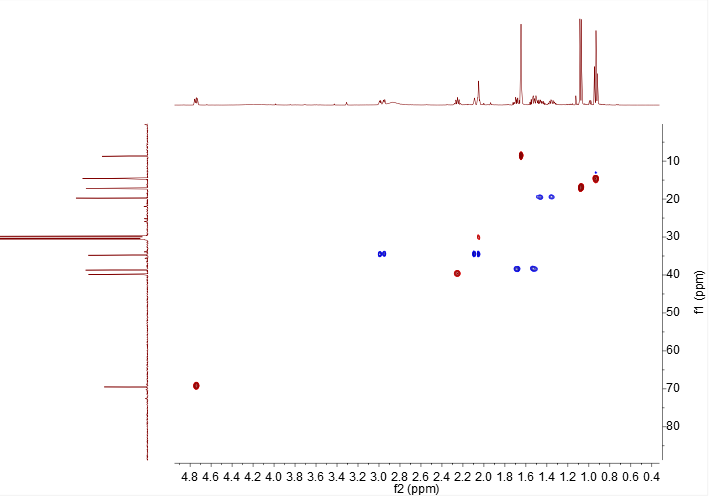
**

**Supplementary Figure 16**. HSQC spectrum of **3** in acetone-*d*_6_

**
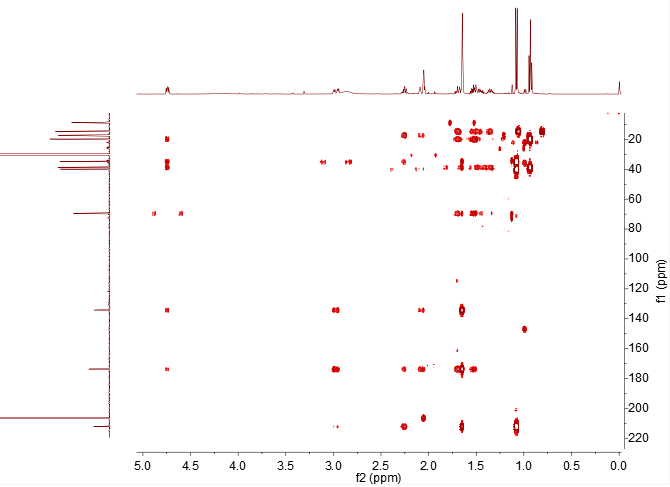
**

**Supplementary Figure 17**. HMBC spectrum of **3** in acetone-*d*_6_


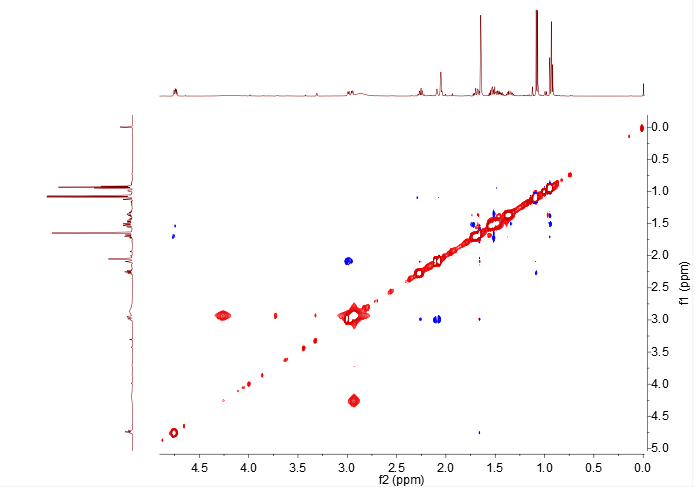


**Supplementary Figure 18**. NOESY spectrum of **3** in acetone-*d*_6_

**
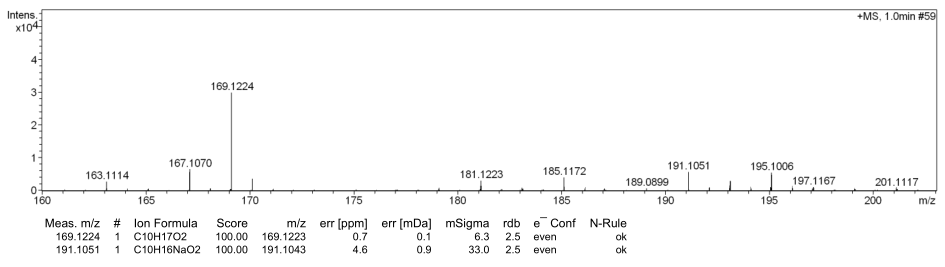
**

**Supplementary Figure 19**. HRESIMS spectrum of **5**

**Supplementary Figure 20.** IR spectrum of **5**

**

**

**Supplementary Figure 21**. UV spectrum of **5**

**
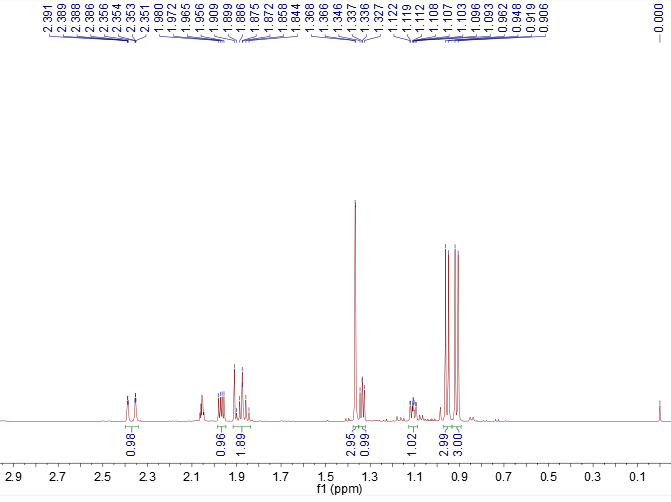
**

**Supplementary Figure 22**. ^1^H NMR spectrum (500 MHz) of **5** in acetone-*d*_6_


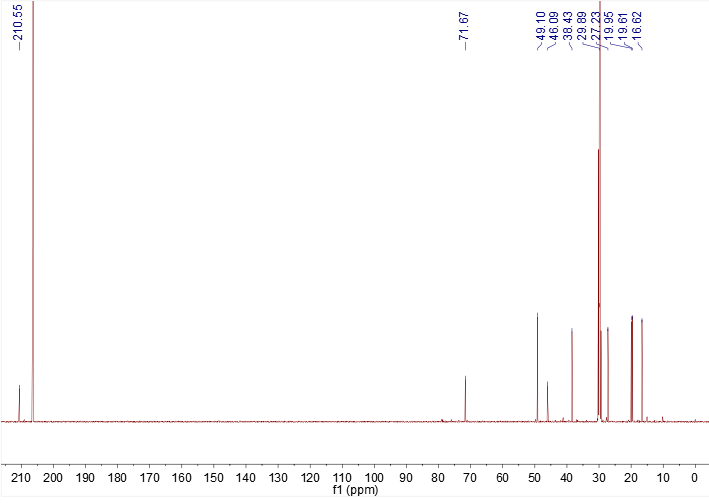


**Supplementary Figure 23**. ^13^C NMR spectrum (125 MHz) of **5** in acetone-*d*_6_

**
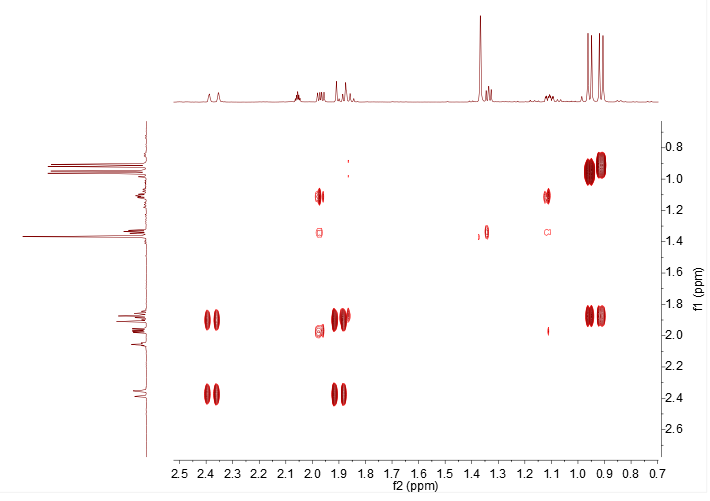
**

**Supplementary Figure 24**. ^1^H-^1^H COSY spectrum of **5** in acetone-*d*_6_

**
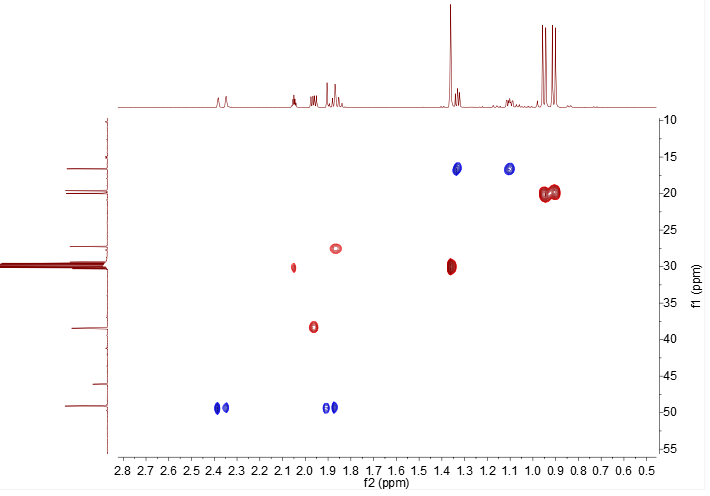
**

**Supplementary Figure 25**. HSQC spectrum of **5** in acetone-*d*_6_

**
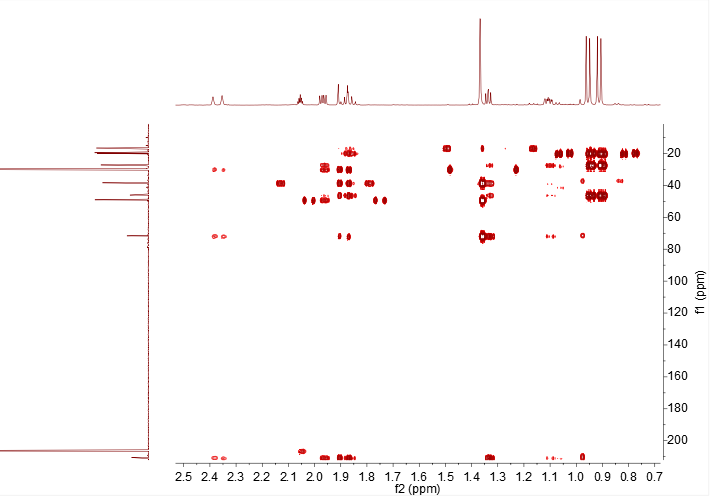
**

**Supplementary Figure 26**. HMBC spectrum of **5** in acetone-*d*_6_

**
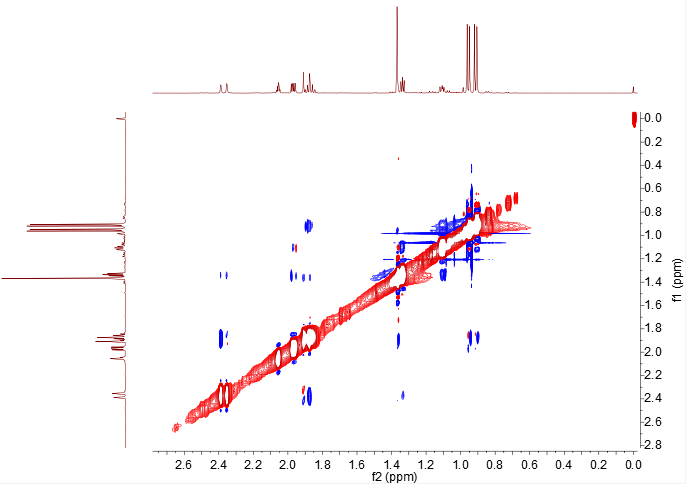
**

**Supplementary Figure 27**. NOESY spectrum of **5** in acetone-*d*_6_


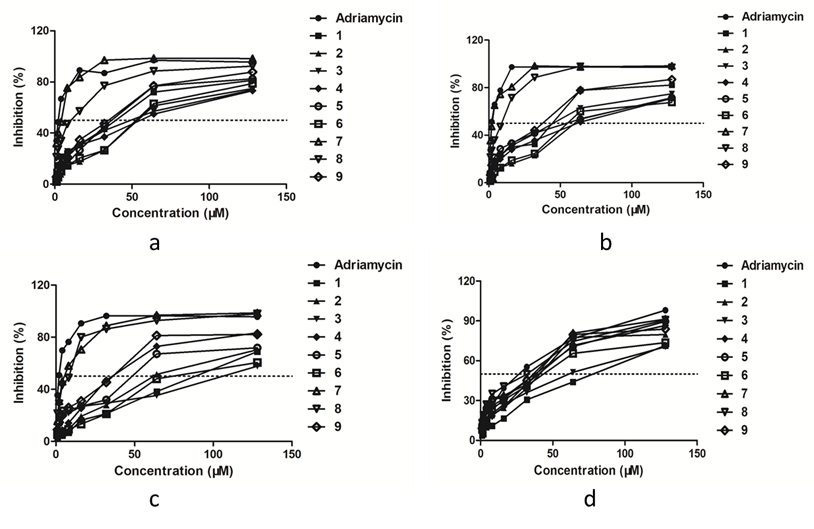


**Supplementary Figure 28**. Dose-response curves for three human cancer cell lines (SF-268, MCF-7, and HepG2) and a normal cell line (LX-2) exposed to compounds **1-9**. a) Dose-response curves of all compounds on HepG2 cells; b) Dose-response curves of all compounds on MCF cells; c) Dose-response curves of all compounds on SF268 cells; d) Dose-response curves of all compounds on LX-2 cells.

**Supplementary Data 1.** Cartesian Coordinates of stable conformers for diaportone A (**1**)

3S,4S,5S,10R-C26 , Δ G = 0.0000 kcal/mol, population = 39.72 %

O -2.656945 1.135946 -1.990768

C -2.468556 2.414789 -1.588324

C -2.729516 2.526904 -0.097447

C -3.579309 1.290315 0.169042

C -3.015697 0.272810 -0.876721

O -2.149717 3.281771 -2.359739

C -3.309021 3.863000 0.339681

O -3.482067 0.885202 1.507756

C -4.062626 -0.718074 -1.369857

C -1.794887 -0.432049 -0.350586

C 1.849176 -1.052214 -0.813365

C 0.582472 -1.071778 -0.340309

C -0.563191 -0.362107 -0.886277

C 2.890085 -1.903228 -0.123445

O 3.950714 -1.005330 0.313868

C 4.642249 -1.333442 1.416798

C 2.313992 -0.257232 -2.001414

C 3.495856 -2.992011 -1.003558

C 5.704619 -0.308238 1.715593

O 4.429559 -2.330970 2.070308

H -1.753947 2.360876 0.396091

H -4.629164 1.506213 -0.105178

H -3.459996 3.868218 1.428844

H -2.628709 4.685893 0.076647

H -4.279417 4.050700 -0.146009

H -4.217813 0.292395 1.712592

H -3.643298 -1.365347 -2.153472

H -4.394667 -1.355770 -0.536731

H -4.935247 -0.182423 -1.772870

H -1.971900 -1.042643 0.539354

H 0.378733 -1.686370 0.544177

H -0.416566 0.253144 -1.777488

H 2.455444 -2.362757 0.774173

H 1.563605 0.462200 -2.351633

H 3.232063 0.295769 -1.748000

H 2.566248 -0.919679 -2.846969

H 4.205206 -3.595676 -0.419290

H 2.701825 -3.654662 -1.379282

H 4.027619 -2.558480 -1.863199

H 5.256027 0.694557 1.772300

H 6.206218 -0.554494 2.658678

H 6.439697 -0.292417 0.896028

3S,4S,5S,10R-C6 , Δ G = 0.0828 kcal/mol, population = 34.53 %

O -1.787437 2.261572 -1.502006

C -2.445600 2.678178 -0.395378

C -3.258863 1.533205 0.180031

C -3.377611 0.596509 -1.016248

C -2.030323 0.851852 -1.768165

O -2.357420 3.809076 0.007328

C -4.556400 1.960739 0.847780

O -3.590589 -0.727456 -0.606191

C -2.146193 0.674531 -3.276786

C -0.917221 0.016118 -1.196079

C 2.402921 0.054686 0.489083

C 1.254772 -0.353403 -0.093833

C 0.203217 0.496377 -0.627346

C 3.404034 -0.959198 1.014259

O 3.299638 -2.221703 0.324710

C 3.846936 -2.303075 -0.902910

C 2.810134 1.484690 0.699778

C 3.212245 -1.264231 2.495116

C 3.577972 -3.644731 -1.529459

O 4.454050 -1.395106 -1.422960

H -2.603261 1.034418 0.917716

H -4.198955 0.946250 -1.669751

H -5.079535 1.081679 1.251201

H -4.355470 2.658187 1.673847

H -5.223993 2.462240 0.129661

H -3.924201 -1.240221 -1.354840

H -1.197794 0.931124 -3.770316

H -2.384600 -0.373756 -3.512029

H -2.946267 1.315616 -3.676613

H -1.074263 -1.063532 -1.272653

H 1.090008 -1.431381 -0.190233

H 0.331299 1.580133 -0.567905

H 4.417108 -0.564167 0.840269

H 2.016969 2.199993 0.450387

H 3.107440 1.661515 1.747073

H 3.693411 1.719507 0.080176

H 3.988648 -1.959496 2.847777

H 3.278977 -0.340139 3.086981

H 2.222991 -1.715013 2.664915

H 2.528512 -3.674118 -1.864753

H 4.234480 -3.790899 -2.395358

H 3.714464 -4.452119 -0.796785

3S,4S,5S,10R-C41 , Δ G = 1.0065 kcal/mol, population = 7.25 %

O -1.921465 2.191438 -1.535536

C -2.428521 2.887662 -0.491101

C -3.331371 1.989452 0.334474

C -3.668677 0.882803 -0.657326

C -2.365956 0.806661 -1.519247

O -2.163998 4.048330 -0.314108

C -4.503505 2.707029 0.985197

O -4.017350 -0.304106 0.002772

C -2.626178 0.381185 -2.958492

C -1.328218 -0.065231 -0.865241

C 2.094318 -0.209669 0.592984

C 0.859188 -0.546115 0.158190

C -0.113068 0.343037 -0.458014

C 3.005842 -1.222968 1.254256

O 4.275725 -1.197289 0.537578

C 5.371646 -0.735083 1.163814

C 2.683527 1.172134 0.491542

C 2.539543 -2.666566 1.292943

C 6.566148 -0.767601 0.247365

O 5.381613 -0.331591 2.305527

H -2.686264 1.540962 1.112678

H -4.488189 1.225187 -1.316901

H -5.102538 1.994203 1.570125

H -4.147250 3.500166 1.658417

H -5.155269 3.167873 0.226413

H -4.479177 -0.885134 -0.616728

H -1.695688 0.407675 -3.543582

H -3.017000 -0.647321 -2.978243

H -3.365753 1.048177 -3.426512

H -1.629435 -1.108531 -0.734211

H 0.528415 -1.581901 0.271727

H 0.158484 1.391113 -0.604899

H 3.218178 -0.875849 2.277547

H 1.939940 1.937771 0.239257

H 3.164827 1.458090 1.440838

H 3.472361 1.199894 -0.279149

H 3.306618 -3.286500 1.779021

H 1.610266 -2.758707 1.872911

H 2.364502 -3.058400 0.279960

H 6.436670 -0.007018 -0.538886

H 7.478858 -0.552543 0.815308

H 6.641489 -1.745108 -0.249847

3S,4S,5S,10R-C36 , Δ G = 1.2155 kcal/mol, population = 5.09 %

O -3.252040 2.285631 0.268342

C -4.429708 1.680007 0.012068

C -4.310784 0.817161 -1.231795

C -3.082499 1.396443 -1.922247

C -2.226835 1.948729 -0.730479

O -5.396624 1.844010 0.711562

C -5.585099 0.743527 -2.060041

O -2.447480 0.419316 -2.701622

C -1.501836 3.236482 -1.078965

C -1.358693 0.871105 -0.142963

C 2.098661 -0.259732 0.651743

C 0.754159 -0.197534 0.525196

C -0.017117 0.896026 -0.041499

C 2.715497 -1.513395 1.236637

O 3.455737 -1.167701 2.441313

C 2.780294 -1.107846 3.602977

C 3.056594 0.826419 0.247795

C 3.704877 -2.211501 0.315870

C 3.676653 -0.656317 4.726215

O 1.604889 -1.374139 3.711936

H -4.042931 -0.196023 -0.879168

H -3.391469 2.259816 -2.542024

H -5.435489 0.083557 -2.926752

H -6.414916 0.347577 -1.456935

H -5.873260 1.740602 -2.428405

H -1.744323 0.836358 -3.218439

H -0.956211 3.634723 -0.212280

H -0.783927 3.063586 -1.894252

H -2.229149 3.989744 -1.413311

H -1.895578 -0.013713 0.216855

H 0.170275 -1.054255 0.878959

H 0.532304 1.767197 -0.405957

H 1.913169 -2.204685 1.525566

H 2.558195 1.785710 0.060140

H 3.809448 0.978687 1.037120

H 3.607635 0.549815 -0.667362

H 4.085026 -3.126121 0.794655

H 3.207029 -2.488398 -0.625239

H 4.561587 -1.562934 0.081066

H 3.904752 0.413572 4.595591

H 3.172101 -0.804594 5.688247

H 4.630646 -1.201875 4.701773

3S,4S,5S,10R-C37 , Δ G = 1.3052 kcal/mol, population = 4.38 %

O -2.492524 3.094205 -0.242246

C -3.653871 2.955842 0.429473

C -4.210296 1.557656 0.224925

C -3.482418 1.089397 -1.029048

C -2.125481 1.871998 -0.971609

O -4.125205 3.853929 1.079386

C -5.730366 1.494226 0.193141

O -3.356991 -0.306989 -1.034563

C -1.639325 2.299672 -2.345157

C -1.111660 1.136434 -0.140166

C 2.237069 -0.491233 -0.031659

C 1.018551 -0.012652 0.307905

C 0.093648 0.704865 -0.553870

C 3.028491 -1.250702 1.008315

O 4.295917 -0.551783 1.168772

C 4.892503 -0.591924 2.371336

C 2.874668 -0.349520 -1.385096

C 3.307078 -2.706453 0.647633

C 6.186196 0.179351 2.360144

O 4.435266 -1.177933 3.327609

H -3.835868 0.949863 1.069465

H -4.036331 1.442356 -1.920002

H -6.062756 0.453866 0.065438

H -6.152658 1.884314 1.130521

H -6.132466 2.092013 -0.639804

H -2.992506 -0.587609 -1.885416

H -0.719970 2.897475 -2.275125

H -1.436221 1.418633 -2.971632

H -2.414565 2.902616 -2.838730

H -1.416113 0.936881 0.893298

H 0.673992 -0.179608 1.334876

H 0.403590 0.890493 -1.584717

H 2.505055 -1.211007 1.972727

H 2.333734 0.342822 -2.041679

H 3.909384 0.011841 -1.277531

H 2.931213 -1.324750 -1.897856

H 3.838935 -3.201443 1.472840

H 2.359129 -3.238212 0.475963

H 3.921647 -2.781266 -0.261461

H 5.999271 1.218822 2.051393

H 6.642553 0.157580 3.356567

H 6.873848 -0.262441 1.622891

3S,4S,5S,10R-C14 , Δ G = 1.6491 kcal/mol, population = 2.45 %

O -1.978093 2.796316 -0.364612

C -2.950552 3.005182 0.545549

C -3.884770 1.808700 0.593184

C -3.611678 1.120243 -0.738322

C -2.121185 1.497876 -1.041101

O -3.010029 4.018616 1.194414

C -5.334229 2.164456 0.889757

O -3.864198 -0.255738 -0.643891

C -1.869037 1.736710 -2.519338

C -1.172912 0.535662 -0.382752

C 1.553650 -1.955116 -0.850060

C 0.622940 -1.145344 -0.299565

C -0.252981 -0.225061 -1.004162

C 2.410959 -2.849144 0.028548

O 1.750181 -3.173345 1.268354

C 0.790345 -4.117445 1.227709

C 1.860910 -2.058881 -2.315844

C 3.738119 -2.202374 0.407069

C 0.133695 -4.283453 2.571445

O 0.497416 -4.725070 0.223528

H -3.499358 1.147861 1.391765

H -4.241914 1.586795 -1.519500

H -5.945298 1.251427 0.935149

H -5.414281 2.688197 1.853317

H -5.747075 2.819843 0.106953

H -3.784647 -0.652048 -1.522805

H -0.833471 2.055936 -2.701275

H -2.057577 0.818151 -3.094363

H -2.548309 2.518821 -2.886880

H -1.271753 0.456515 0.705642

H 0.496588 -1.183174 0.787065

H -0.153139 -0.162204 -2.090213

H 2.592227 -3.790896 -0.512230

H 1.330524 -1.315107 -2.922757

H 2.941291 -1.940004 -2.503157

H 1.588176 -3.062903 -2.685307

H 4.350490 -2.900429 0.997203

H 4.300089 -1.926286 -0.496616

H 3.561773 -1.291469 0.998452

H -0.552890 -3.437126 2.735938

H -0.439608 -5.217821 2.593867

H 0.881178 -4.265712 3.376521

3S,4S,5S,10R-C21 , Δ G = 1.9447 kcal/mol, population = 1.49 %

O -2.884539 0.933307 -1.263696

C -3.199592 1.981476 -2.054389

C -2.736672 3.285014 -1.417687

C -1.827050 2.815512 -0.279320

C -2.335026 1.359263 0.014766

O -3.777072 1.836942 -3.101112

C -3.908774 4.202028 -1.055293

O -0.511078 2.831328 -0.784990

C -3.455654 1.336206 1.052566

C -1.223403 0.431819 0.415635

C 1.301104 -2.014647 -0.821856

C 0.559376 -1.215400 -0.021282

C -0.525315 -0.343045 -0.436061

C 2.358763 -2.889195 -0.187880

O 3.623656 -2.554613 -0.825196

C 4.747453 -2.658721 -0.096879

C 1.148486 -2.122432 -2.312613

C 2.107190 -4.385913 -0.342111

C 5.956939 -2.280730 -0.910946

O 4.765854 -3.013903 1.060789

H -2.106436 3.799360 -2.158485

H -1.920304 3.434524 0.630403

H -3.534157 5.149872 -0.642123

H -4.497396 4.425742 -1.956838

H -4.583345 3.749058 -0.315103

H 0.039177 2.258407 -0.229968

H -3.860562 0.317809 1.140939

H -3.068476 1.646315 2.034092

H -4.275136 2.012570 0.775370

H -0.977067 0.433081 1.482697

H 0.782064 -1.218174 1.051929

H -0.802516 -0.322789 -1.492714

H 2.449889 -2.645247 0.878722

H 0.755822 -3.112723 -2.599011

H 0.476326 -1.361921 -2.727816

H 2.131673 -2.020075 -2.797846

H 2.888907 -4.953807 0.182844

H 1.132513 -4.648944 0.095510

H 2.108567 -4.681929 -1.401362

H 5.839031 -1.260095 -1.304825

H 6.858775 -2.344907 -0.291214

H 6.046617 -2.956485 -1.775186

3S,4S,5S,10R-C4 , Δ G = 2.0570 kcal/mol, population = 1.23 %

O -2.022320 1.763619 -1.550984

C -2.506087 3.017414 -1.421148

C -3.211030 3.178645 -0.081080

C -2.796028 1.921161 0.687003

C -2.431729 0.902827 -0.451666

O -2.374005 3.842568 -2.288169

C -4.710297 3.446848 -0.243442

O -1.675418 2.278006 1.464589

C -3.638927 0.083628 -0.904404

C -1.292504 0.000695 -0.071531

C 2.402970 -0.456287 -0.186352

C 1.085513 -0.638460 0.057177

C 0.003443 0.241443 -0.346802

C 3.383581 -1.504384 0.297989

O 4.306211 -0.894409 1.244244

C 3.922273 -0.803533 2.530308

C 2.992061 0.723904 -0.907320

C 4.241529 -2.112914 -0.800445

C 4.950207 -0.068964 3.350366

O 2.879196 -1.248026 2.953233

H -2.753724 4.043750 0.421905

H -3.608990 1.509207 1.310643

H -5.175732 3.602605 0.740715

H -4.861896 4.354320 -0.845786

H -5.233821 2.619040 -0.742185

H -1.200029 1.470010 1.709925

H -3.368615 -0.524622 -1.779538

H -3.963877 -0.588529 -0.096917

H -4.484548 0.728646 -1.177870

H -1.575261 -0.894746 0.492043

H 0.789505 -1.532094 0.617346

H 0.248879 1.140673 -0.916905

H 2.831632 -2.290192 0.830061

H 2.277347 1.546988 -1.028818

H 3.866553 1.104902 -0.356614

H 3.349385 0.438065 -1.911253

H 4.905062 -2.882298 -0.378572

H 3.598057 -2.581428 -1.559806

H 4.863820 -1.350474 -1.291267

H 4.924040 0.999791 3.084002

H 4.725794 -0.181873 4.417589

H 5.960390 -0.439683 3.125988

3S,4S,5S,10R-C16 , Δ G = 2.0921 kcal/mol, population = 1.16 %

O -1.886314 1.959621 -1.799417

C -2.220823 3.266573 -1.845523

C -2.814429 3.710807 -0.515124

C -2.492827 2.542543 0.420374

C -2.314325 1.328760 -0.559745

O -2.056192 3.930414 -2.836741

C -4.281761 4.129947 -0.645520

O -1.294101 2.875067 1.084227

C -3.630499 0.598611 -0.820771

C -1.257412 0.366260 -0.097881

C 2.348403 -0.539303 -0.330838

C 1.041692 -0.519560 0.012644

C 0.036783 0.410644 -0.468714

C 3.287206 -1.565347 0.279354

O 2.584919 -2.755962 0.689079

C 2.242670 -3.633644 -0.273996

C 3.010906 0.407778 -1.288299

C 4.002460 -1.042188 1.519424

C 1.444833 -4.777132 0.291816

O 2.533338 -3.489029 -1.439173

H -2.230973 4.580266 -0.177171

H -3.305736 2.326878 1.136267

H -4.658827 4.493083 0.321641

H -4.371821 4.943496 -1.379918

H -4.927541 3.303806 -0.975280

H -0.885855 2.057252 1.406883

H -3.486151 -0.159233 -1.604148

H -3.971528 0.095185 0.095575

H -4.418299 1.290483 -1.147527

H -1.598802 -0.397685 0.608670

H 0.689724 -1.280470 0.716441

H 0.337778 1.183073 -1.180716

H 4.023715 -1.855068 -0.485998

H 2.352482 1.220802 -1.617045

H 3.910266 0.860362 -0.838191

H 3.352086 -0.142915 -2.182244

H 4.710963 -1.793814 1.898237

H 4.562219 -0.126576 1.280826

H 3.272280 -0.807843 2.308403

H 0.422891 -4.424056 0.505237

H 1.396191 -5.594850 -0.436951

H 1.879940 -5.125275 1.238831

3S,4S,5S,10R-C1 , Δ G = 2.4630 kcal/mol, population = 0.62 %

O -2.393266 1.271475 -1.637723

C -2.531688 2.576123 -1.949564

C -2.802927 3.392765 -0.697081

C -2.572571 2.407744 0.463330

C -2.635630 0.998606 -0.230490

O -2.453528 2.969452 -3.085110

C -4.184992 4.053612 -0.756503

O -1.365621 2.642005 1.152034

C -4.022376 0.366760 -0.108753

C -1.569154 0.073119 0.279714

C 1.570527 -1.746361 -0.559048

C 0.494200 -1.262951 0.100821

C -0.517074 -0.366641 -0.434307

C 2.501846 -2.697061 0.163352

O 3.832122 -2.107623 0.226887

C 4.099421 -1.258166 1.234398

C 1.931497 -1.411667 -1.980084

C 2.669165 -4.047326 -0.516476

C 5.479669 -0.671035 1.094287

O 3.318515 -1.000347 2.122324

H -2.037235 4.181809 -0.639851

H -3.364808 2.482046 1.220631

H -4.346266 4.678750 0.133728

H -4.260157 4.691190 -1.649239

H -4.992013 3.307067 -0.802899

H -0.623952 2.402747 0.575864

H -4.076048 -0.540327 -0.727603

H -4.223026 0.093276 0.937276

H -4.804972 1.065256 -0.439970

H -1.695750 -0.239082 1.321129

H 0.358012 -1.567565 1.144357

H -0.425379 -0.038441 -1.472152

H 2.147417 -2.830465 1.193855

H 1.377985 -0.546164 -2.364694

H 3.008363 -1.193113 -2.055928

H 1.732019 -2.262854 -2.653406

H 3.336426 -4.689452 0.077443

H 1.691419 -4.543572 -0.605964

H 3.099320 -3.938047 -1.522782

H 5.479439 0.049425 0.260691

H 5.760588 -0.153846 2.019364

H 6.210822 -1.455190 0.851972

3S,4S,5S,10R-C42 , Δ G = 2.7767 kcal/mol, population = 0.36 %

O -2.640447 2.202891 0.517280

C -3.974071 2.377113 0.576106

C -4.675015 1.482819 -0.436404

C -3.571739 0.556523 -0.950139

C -2.242848 1.304790 -0.569320

O -4.483755 3.158589 1.338792

C -5.426264 2.302527 -1.489958

O -3.715456 -0.685011 -0.294490

C -1.737990 2.163341 -1.721528

C -1.221876 0.370445 0.012430

C 2.011495 -1.408662 -0.476292

C 0.850451 -0.954511 0.046474

C -0.070016 -0.013229 -0.569054

C 2.814341 -2.424642 0.310221

O 4.133610 -1.880015 0.593502

C 4.275058 -1.098746 1.679518

C 2.585684 -0.990491 -1.801146

C 3.045529 -3.738113 -0.421571

C 5.676935 -0.556183 1.775048

O 3.380781 -0.864880 2.460337

H -5.399860 0.869411 0.120314

H -3.611201 0.429268 -2.045404

H -5.973527 1.636064 -2.172335

H -6.150116 2.969009 -0.999002

H -4.745553 2.924154 -2.090353

H -3.014358 -1.275757 -0.606089

H -0.874969 2.764720 -1.403914

H -1.439680 1.535185 -2.572509

H -2.528121 2.844381 -2.067171

H -1.491916 -0.036347 0.991707

H 0.555653 -1.327639 1.033271

H 0.194342 0.385339 -1.551382

H 2.315402 -2.606954 1.271005

H 2.090072 -0.106133 -2.220216

H 3.658465 -0.765516 -1.693382

H 2.505705 -1.804055 -2.542154

H 3.607733 -4.433151 0.219535

H 2.079403 -4.198252 -0.676544

H 3.615829 -3.584432 -1.349285

H 5.828483 0.190673 0.979505

H 5.827285 -0.082077 2.752089

H 6.411988 -1.358360 1.617768

3S,4S,5S,10R-C44 , Δ G = 2.8878 kcal/mol, population = 0.30 %

O -2.006982 2.155180 -1.728568

C -2.358822 2.876864 -0.638714

C -3.231345 2.035235 0.274554

C -3.738909 0.959042 -0.677305

C -2.536887 0.801730 -1.665534

O -2.001355 4.016520 -0.490063

C -4.281708 2.824183 1.040396

O -4.097583 -0.205653 0.016210

C -2.968117 0.401518 -3.070779

C -1.497600 -0.137460 -1.115839

C 2.023796 -0.486646 0.040088

C 0.740123 -0.751945 -0.289167

C -0.228611 0.194786 -0.817447

C 2.910781 -1.545662 0.667467

O 4.270716 -1.467097 0.146432

C 4.497469 -1.675402 -1.160540

C 2.671185 0.864425 -0.108739

C 2.408691 -2.981284 0.656888

C 5.960329 -1.507811 -1.482559

O 3.633931 -1.948212 -1.963374

H -2.542407 1.540491 0.984083

H -4.595949 1.358812 -1.251327

H -4.863052 2.148629 1.684413

H -3.807667 3.588855 1.672698

H -4.976400 3.330219 0.351859

H -4.658804 -0.750761 -0.551699

H -2.101866 0.370227 -3.747136

H -3.425587 -0.599231 -3.049922

H -3.705885 1.117919 -3.462319

H -1.847577 -1.162218 -0.961960

H 0.364377 -1.769484 -0.154160

H 0.089884 1.226211 -0.986076

H 3.070801 -1.241480 1.715691

H 1.939239 1.670703 -0.241414

H 3.288816 1.103203 0.771873

H 3.344897 0.883778 -0.982423

H 3.179666 -3.637610 1.086191

H 1.506242 -3.072677 1.277886

H 2.179399 -3.320043 -0.361398

H 6.565432 -2.167654 -0.843287

H 6.137701 -1.736874 -2.539762

H 6.266887 -0.472526 -1.267641

3S,4S,5S,10R-C35 , Δ G = 2.9681 kcal/mol, population = 0.26 %

O -3.176134 1.893886 0.658795

C -4.478689 1.768184 0.341873

C -4.634970 1.124901 -1.028734

C -3.227550 0.640023 -1.379272

C -2.293606 1.478478 -0.433350

O -5.354940 2.145915 1.077777

C -5.297149 2.080000 -2.026876

O -3.176427 -0.746301 -1.118568

C -1.782466 2.735981 -1.123786

C -1.224449 0.629644 0.192155

C 2.393482 -0.247808 0.297642

C 1.063398 -0.206506 0.538956

C 0.091343 0.673104 -0.088772

C 3.224876 -1.278492 1.027033

O 4.275766 -0.558174 1.730242

C 4.727084 -1.068803 2.887885

C 3.126018 0.638475 -0.669549

C 3.863713 -2.324365 0.118492

C 5.814745 -0.206528 3.472529

O 4.305278 -2.092408 3.378785

H -5.277348 0.240175 -0.902218

H -2.961434 0.852316 -2.429066

H -5.450443 1.576011 -2.992207

H -6.275684 2.403053 -1.643133

H -4.687514 2.978758 -2.202203

H -2.284714 -1.061788 -1.326045

H -1.220788 3.361218 -0.416069

H -1.127007 2.476926 -1.966856

H -2.620669 3.326067 -1.519649

H -1.596889 -0.098035 0.919548

H 0.656817 -0.907575 1.276755

H 0.459136 1.393061 -0.823641

H 2.607712 -1.783826 1.781508

H 3.495859 0.057991 -1.531781

H 2.504989 1.455362 -1.057080

H 4.010597 1.076090 -0.181087

H 4.406053 -3.065527 0.723253

H 3.084637 -2.847268 -0.456222

H 4.569948 -1.861728 -0.586375

H 5.454076 0.826329 3.588405

H 6.129749 -0.609534 4.442014

H 6.672463 -0.178609 2.782982

3S,4S,5S,10R-C12 , Δ G = 2.9750 kcal/mol, population = 0.26 %

O -2.795871 1.662731 0.316960

C -4.100849 1.780837 0.010150

C -4.355157 1.336256 -1.423035

C -3.037280 0.693979 -1.858975

C -1.985436 1.255370 -0.835190

O -4.908579 2.199766 0.800576

C -4.874799 2.488630 -2.288263

O -3.196432 -0.706320 -1.777934

C -1.290960 2.497641 -1.376704

C -1.058173 0.184767 -0.337949

C 2.424137 -1.128537 -0.272544

C 1.095675 -0.978243 -0.077999

C 0.265228 0.105882 -0.573220

C 3.159638 -2.311831 0.331910

O 2.286129 -3.442443 0.525815

C 1.971202 -4.171624 -0.562046

C 3.303723 -0.189029 -1.044912

C 3.759935 -1.995162 1.696398

C 0.989565 -5.260031 -0.221612

O 2.416972 -3.947634 -1.664110

H -5.120798 0.546266 -1.386608

H -2.743550 0.996800 -2.878593

H -5.108416 2.129251 -3.300943

H -5.791765 2.904309 -1.845995

H -4.140046 3.302898 -2.374153

H -2.363805 -1.122764 -2.044574

H -0.646586 2.945102 -0.607375

H -0.676963 2.247177 -2.253004

H -2.030940 3.247778 -1.688150

H -1.548379 -0.596624 0.250607

H 0.576259 -1.748109 0.501193

H 0.750026 0.884807 -1.166538

H 3.953789 -2.616208 -0.367238

H 2.785885 0.722241 -1.367676

H 4.178948 0.113740 -0.445863

H 3.698923 -0.697799 -1.941404

H 4.326945 -2.858558 2.075220

H 4.442448 -1.136358 1.623462

H 2.963218 -1.746568 2.413304

H -0.005322 -4.807635 -0.079845

H 0.939317 -5.986981 -1.040820

H 1.266015 -5.753924 0.720205

3S,4S,5S,10R-C33 , Δ G = 2.9819 kcal/mol, population = 0.26 %

O -2.401411 2.449724 0.448930

C -3.728228 2.628577 0.591990

C -4.496462 1.648100 -0.282837

C -3.429625 0.671041 -0.778832

C -2.080072 1.449065 -0.570668

O -4.184480 3.475780 1.317334

C -5.312487 2.372656 -1.358065

O -3.521073 -0.496693 0.008929

C -1.660503 2.188737 -1.834062

C -1.015516 0.572601 0.023834

C 2.168801 -1.276594 -0.533832

C 1.055743 -0.755347 0.030486

C 0.084021 0.118290 -0.606040

C 3.025139 -2.208590 0.292966

O 4.369470 -1.651429 0.307688

C 5.129447 -1.861681 1.395255

C 2.622283 -1.027801 -1.944509

C 3.087067 -3.637701 -0.237288

C 6.486907 -1.228529 1.237919

O 4.756005 -2.485618 2.363717

H -5.184552 1.094856 0.374310

H -3.546636 0.432375 -1.849957

H -5.901621 1.649769 -1.940927

H -6.003826 3.085781 -0.886194

H -4.670184 2.932198 -2.054140

H -2.840606 -1.118048 -0.288763

H -0.775903 2.810590 -1.638991

H -1.425464 1.479135 -2.639497

H -2.472254 2.839863 -2.187028

H -1.204453 0.276563 1.060176

H 0.844143 -1.013757 1.074329

H 0.264650 0.406417 -1.644310

H 2.661193 -2.222919 1.328732

H 2.535949 -1.944441 -2.552376

H 2.049478 -0.238024 -2.445818

H 3.686103 -0.743492 -1.951770

H 3.682450 -4.264438 0.442353

H 2.072284 -4.058507 -0.299189

H 3.543732 -3.672113 -1.237243

H 6.378251 -0.158365 1.007253

H 7.069418 -1.361617 2.156896

H 7.013831 -1.694391 0.390980

3S,4S,5S,10R-C20 , Δ G = 2.9920 kcal/mol, population = 0.25 %

O -2.763688 2.159495 0.713975

C -4.108670 2.175964 0.672995

C -4.618640 1.323116 -0.479810

C -3.376617 0.585476 -0.982508

C -2.178996 1.430757 -0.416246

O -4.765158 2.808609 1.461370

C -5.380770 2.162573 -1.509852

O -3.420745 -0.727683 -0.466303

C -1.695313 2.458225 -1.430721

C -1.099418 0.560938 0.159209

C 2.404385 -0.658181 -0.145620

C 1.136222 -0.462407 0.277130

C 0.150654 0.421276 -0.321190

C 3.326046 -1.604377 0.603911

O 2.588996 -2.629434 1.300211

C 2.109594 -3.651085 0.564833

C 3.042742 0.012509 -1.326962

C 4.168489 -0.892851 1.655818

C 1.293445 -4.592356 1.408643

O 2.305898 -3.764082 -0.623493

H -5.305247 0.576701 -0.051938

H -3.317728 0.571826 -2.084302

H -5.791118 1.516352 -2.299330

H -6.214297 2.687684 -1.021258

H -4.735950 2.916968 -1.984600

H -2.633421 -1.201175 -0.772446

H -0.939744 3.117365 -0.981314

H -1.257011 1.960646 -2.307040

H -2.532434 3.078324 -1.780006

H -1.405320 -0.011650 1.040034

H 0.799070 -1.026909 1.152132

H 0.450530 0.990184 -1.204488

H 3.980187 -2.099915 -0.130032

H 2.408444 0.782303 -1.782844

H 3.997561 0.486031 -1.042878

H 3.283511 -0.738734 -2.099206

H 4.857802 -1.601763 2.138273

H 4.762173 -0.092092 1.192112

H 3.519302 -0.445070 2.423006

H 0.319579 -4.122486 1.621728

H 1.127606 -5.530989 0.866845

H 1.787780 -4.781153 2.371641

3S,4S,5S,10R-C48 , Δ G = 3.2016 kcal/mol, population = 0.18 %

O -1.989132 1.343141 -2.004601

C -2.301204 2.580579 -2.445629

C -2.629486 3.495436 -1.273137

C -2.159196 2.690670 -0.058702

C -2.193464 1.206453 -0.570578

O -2.310592 2.853750 -3.618335

C -4.088357 3.961513 -1.292369

O -0.849532 3.126080 0.229243

C -3.550845 0.545487 -0.337794

C -1.089519 0.373189 0.015589

C 2.402585 -0.829940 -0.462331

C 1.174106 -0.602970 0.055442

C 0.115269 0.182313 -0.555252

C 3.447759 -1.622424 0.296633

O 3.938314 -2.664198 -0.595697

C 5.202434 -2.594612 -1.049546

C 2.870510 -0.316435 -1.797272

C 3.015864 -2.297970 1.585294

C 5.504932 -3.746650 -1.970474

O 5.977051 -1.708864 -0.764371

H -1.978741 4.378167 -1.361505

H -2.824840 2.799394 0.815795

H -4.274662 4.656548 -0.460743

H -4.298097 4.486024 -2.235859

H -4.797360 3.126218 -1.205170

H -0.403346 2.436577 0.743763

H -3.570054 -0.444169 -0.816420

H -3.729355 0.417559 0.739772

H -4.369239 1.147959 -0.753963

H -1.309271 -0.070701 0.992438

H 0.928497 -1.030149 1.031163

H 0.295467 0.634623 -1.533049

H 4.300376 -0.954395 0.496479

H 2.202691 0.440741 -2.225216

H 3.878672 0.120052 -1.711534

H 2.949394 -1.145352 -2.520607

H 3.864728 -2.853039 2.009696

H 2.693597 -1.552317 2.325906

H 2.189616 -3.002922 1.410577

H 4.935375 -3.618631 -2.904603

H 6.577026 -3.771845 -2.197936

H 5.183568 -4.694830 -1.516576

3S,4S,5S,10R-C45 , Δ G = 3.2718 kcal/mol, population = 0.16 %

O -1.793211 1.609875 -1.807140

C -1.629357 2.903967 -1.447143

C -2.065428 3.094687 -0.006332

C -2.964786 1.886255 0.219581

C -2.314644 0.808154 -0.712817

O -1.200242 3.726442 -2.213983

C -2.665692 4.459483 0.292772

O -3.014873 1.547325 1.580208

C -3.327127 -0.170641 -1.292211

C -1.199044 0.094796 0.002236

C 1.338469 -0.765252 1.692737

C 1.229338 -0.447458 0.384351

C 0.106157 0.185237 -0.306319

C 2.596035 -1.424268 2.230045

O 3.682610 -1.368407 1.287566

C 4.458334 -0.266187 1.281965

C 0.276316 -0.530683 2.731753

C 2.386963 -2.897540 2.564866

C 5.492147 -0.337498 0.190371

O 4.313791 0.657183 2.049356

H -1.158877 2.941139 0.607796

H -3.976554 2.105110 -0.170784

H -2.944806 4.522630 1.354511

H -1.941929 5.257754 0.073463

H -3.567186 4.636653 -0.314401

H -3.754904 0.943172 1.729940

H -2.837710 -0.863201 -1.992184

H -3.780565 -0.762517 -0.482698

H -4.124811 0.371969 -1.821199

H -1.539404 -0.533005 0.826050

H 2.103313 -0.637835 -0.244638

H 0.377935 0.777836 -1.186864

H 2.906509 -0.875657 3.133167

H -0.367973 0.321994 2.473424

H -0.383939 -1.408548 2.842519

H 0.729449 -0.346736 3.719036

H 3.307508 -3.326883 2.987239

H 1.580191 -3.012152 3.302669

H 2.112894 -3.458501 1.658571

H 5.006048 -0.115211 -0.773456

H 6.277293 0.406041 0.372092

H 5.919652 -1.347183 0.122026

3S,4S,5S,10R-C49 , Δ G = 3.8127 kcal/mol, population = 0.06 %

O -2.328658 2.560353 0.050198

C -3.579340 2.264239 0.458244

C -4.067331 1.001918 -0.231098

C -3.136330 0.891196 -1.431903

C -1.825540 1.602361 -0.942351

O -4.166474 2.944601 1.260279

C -5.560468 0.995013 -0.525360

O -2.977918 -0.452623 -1.806276

C -1.152098 2.407715 -2.039082

C -0.938058 0.620981 -0.230440

C 0.742591 -1.923662 0.572485

C 1.140691 -0.743330 0.048369

C 0.312104 0.281279 -0.586546

C 1.788396 -2.818587 1.206375

O 1.822113 -4.096492 0.511258

C 2.564050 -4.187149 -0.606648

C -0.665568 -2.454203 0.558223

C 1.516534 -3.137982 2.668437

C 2.417054 -5.544123 -1.243937

O 3.245808 -3.284300 -1.036245

H -3.825368 0.166848 0.452147

H -3.549455 1.485831 -2.268322

H -5.848844 0.043742 -0.995483

H -6.136976 1.115930 0.403140

H -5.831298 1.815961 -1.207503

H -2.447869 -0.497738 -2.614465

H -0.268785 2.938600 -1.657697

H -0.835719 1.748996 -2.860835

H -1.860283 3.146115 -2.441071

H -1.396787 0.140982 0.637720

H 2.217450 -0.541008 0.041732

H 0.788242 0.810072 -1.419389

H 2.776036 -2.351966 1.095087

H -1.271780 -1.990660 -0.232069

H -0.653542 -3.544932 0.409521

H -1.179392 -2.267139 1.517006

H 2.317110 -3.775689 3.072080

H 1.480378 -2.206529 3.252500

H 0.558469 -3.664318 2.790916

H 1.410646 -5.626975 -1.684413

H 3.169306 -5.669722 -2.031596

H 2.512039 -6.337157 -0.488666

**Supplementary Data 2.** Cartesian Coordinates of stable conformers for diaportone B (**3**)

5S, 6S-C1 , Δ G = 0.0000 kcal/mol, population = 32.58 %

C -5.429813 1.351416 -0.171160

C -4.988473 0.111142 0.153950

C -3.649078 0.130336 0.845513

C -3.255662 1.615991 0.912627

C -4.442048 2.350263 0.279174

C -5.732865 -1.172188 -0.086115

C -6.565194 -1.332126 2.353583

H -7.442733 -2.274510 0.610247

H -7.657330 -0.525209 0.660076

O -4.812916 -2.245773 0.045307

C -6.698044 1.758884 -0.850053

O -4.544925 3.562347 0.175465

C -2.946797 2.147492 2.312700

H -3.730171 -0.327359 1.845830

H -2.926116 -0.489423 0.292770

H -2.386052 1.810674 0.260840

H -6.138099 -1.141451 -1.117450

C -6.932215 -1.328749 0.870054

C -7.764665 -1.604303 3.259142

H -6.116032 -0.360111 2.620849

H -5.784120 -2.091478 2.523614

H -7.476793 -1.596915 4.322277

H -8.214860 -2.587064 3.040771

H -8.550534 -0.843213 3.119748

H -5.298162 -3.070705 -0.091134

H -7.496292 1.966926 -0.117059

H -6.537220 2.687352 -1.418550

H -7.071605 0.981957 -1.533625

H -2.781435 3.234949 2.278177

H -2.047376 1.670050 2.730470

H -3.787506 1.951479 2.998424

5S, 6S-C2 , Δ G = 0.5202 kcal/mol, population = 13.53 %

C -5.494182 1.238812 0.167499

C -4.876898 0.035847 0.261612

C -3.407360 0.157301 0.582861

C -3.135026 1.669819 0.650684

C -4.508215 2.309554 0.425163

C -5.521339 -1.314820 0.142432

C -6.878014 -1.093640 2.335380

H -4.928342 -2.020485 2.095296

H -6.260751 -2.919586 1.356979

O -4.612988 -2.160385 -0.551278

C -6.925811 1.566554 -0.107871

O -4.742693 3.506505 0.460060

C -2.484500 2.162719 1.943201

H -3.183832 -0.346672 1.539478

H -2.806953 -0.361304 -0.179790

H -2.506376 1.980454 -0.202366

H -6.462885 -1.211914 -0.430401

C -5.865324 -1.900956 1.524677

C -7.232234 -1.757650 3.665013

H -7.794314 -0.950276 1.736137

H -6.475475 -0.084207 2.523461

H -7.959358 -1.156496 4.233089

H -6.336877 -1.885367 4.295825

H -7.671677 -2.756778 3.508709

H -4.986479 -3.052329 -0.557824

H -7.466040 0.729832 -0.572335

H -7.454111 1.829136 0.824214

H -6.992203 2.445665 -0.767371

H -2.420394 3.261285 1.942272

H -1.469114 1.753884 2.058269

H -3.078174 1.856147 2.820088

5S, 6S-C15 , Δ G = 0.5315 kcal/mol, population = 13.27 %

C -5.264470 1.305085 -0.368700

C -4.810725 0.067274 -0.053451

C -3.597559 0.098052 0.840892

C -3.280707 1.591446 1.030509

C -4.403242 2.314495 0.277963

C -5.449203 -1.230994 -0.451521

C -7.342001 -2.863241 0.141301

H -7.356775 -0.715809 0.412869

H -6.270393 -1.573180 1.518138

O -4.452438 -2.242175 -0.407629

C -6.448455 1.699520 -1.191533

O -4.552735 3.525061 0.230901

C -3.174100 2.062656 2.480730

H -3.820253 -0.405724 1.798007

H -2.771864 -0.471185 0.387404

H -2.343630 1.848161 0.505728

H -5.843706 -1.130371 -1.482230

C -6.636693 -1.548505 0.477200

C -8.548599 -3.134751 1.038261

H -6.625235 -3.697747 0.236300

H -7.659909 -2.844473 -0.916785

H -9.034324 -4.089263 0.781813

H -9.302481 -2.336207 0.940897

H -8.251630 -3.182581 2.098927

H -4.834115 -3.056452 -0.759791

H -7.332135 1.877919 -0.554765

H -6.243704 2.641803 -1.722089

H -6.721083 0.927639 -1.926193

H -3.051543 3.155687 2.516755

H -2.315236 1.597762 2.988355

H -4.085718 1.802658 3.043569

5S, 6S-C4 , Δ G = 0.7831 kcal/mol, population = 8.67 %

C -5.450762 1.196452 0.308094

C -4.749381 0.037834 0.249386

C -3.270675 0.236183 0.465569

C -3.093970 1.754833 0.637320

C -4.520773 2.311185 0.572408

C -5.331213 -1.333806 0.075239

C -6.439876 -3.275077 1.371637

H -6.617597 -1.156844 1.797651

H -5.018362 -1.833914 2.148159

O -4.322676 -2.174854 -0.466347

C -6.922964 1.421072 0.178907

O -4.826149 3.483625 0.721719

C -2.374148 2.191341 1.913264

H -2.938835 -0.325572 1.356360

H -2.701502 -0.179005 -0.380159

H -2.558742 2.174829 -0.232407

H -6.184542 -1.262766 -0.625899

C -5.852903 -1.860272 1.428041

C -7.680819 -3.409538 0.487990

H -6.688126 -3.583023 2.400927

H -5.659159 -3.981574 1.036748

H -8.096810 -4.427648 0.541305

H -7.461168 -3.202993 -0.572173

H -8.469301 -2.706470 0.803841

H -4.716264 -3.036523 -0.654766

H -7.404309 1.474084 1.170658

H -7.117270 2.382962 -0.319827

H -7.418964 0.619197 -0.387077

H -2.388821 3.288146 2.001588

H -1.326284 1.854571 1.914637

H -2.869807 1.770595 2.803616

5S, 6S-C5 , Δ G = 0.9375 kcal/mol, population = 6.68 %

C -5.435050 1.212349 0.377730

C -4.762969 0.037621 0.304945

C -3.265984 0.216941 0.381675

C -3.046864 1.736211 0.475474

C -4.462525 2.317620 0.521607

C -5.357749 -1.337988 0.211728

C -6.137710 -1.415639 2.696966

H -4.264716 -2.147431 1.875720

H -5.682940 -3.091836 1.405426

O -4.598565 -2.059482 -0.752122

C -6.904226 1.485860 0.352447

O -4.735249 3.499087 0.656121

C -2.199032 2.206856 1.657089

H -2.862774 -0.311553 1.263043

H -2.783608 -0.241521 -0.494706

H -2.591823 2.111405 -0.458148

H -6.408113 -1.252213 -0.120997

C -5.321124 -2.060756 1.573650

C -7.652736 -1.489415 2.499520

H -5.825098 -0.364264 2.821278

H -5.875257 -1.919447 3.642117

H -8.185166 -1.046768 3.355989

H -7.987416 -2.535436 2.401167

H -7.981304 -0.951445 1.597668

H -4.917596 -2.972371 -0.754620

H -7.469423 0.688295 -0.150030

H -7.306432 1.588291 1.374470

H -7.098823 2.439128 -0.161987

H -2.182059 3.306299 1.699725

H -1.163169 1.844694 1.572255

H -2.614669 1.834598 2.607873

5S, 6S-C6 , Δ G = 1.0410 kcal/mol, population = 5.61 %

C -5.477265 1.339274 -0.069517

C -4.989854 0.112878 0.242506

C -3.608013 0.169978 0.842652

C -3.233943 1.661866 0.843751

C -4.480029 2.364067 0.294606

C -5.725246 -1.186800 0.074735

C -6.465306 -1.191507 2.590216

H -7.321497 -2.333826 0.950028

H -7.630168 -0.602264 0.868909

O -4.775193 -2.241730 0.122724

C -6.799314 1.711517 -0.660180

O -4.615982 3.572238 0.184147

C -2.812530 2.229796 2.199062

H -3.616444 -0.257353 1.859793

H -2.912378 -0.456349 0.263218

H -2.427029 1.851292 0.114136

H -6.214320 -1.170184 -0.920313

C -6.854747 -1.344997 1.115821

C -5.567278 -2.297121 3.148379

H -7.396996 -1.155426 3.180253

H -5.986331 -0.207566 2.737930

H -5.368237 -2.139779 4.220530

H -4.605115 -2.343426 2.619883

H -6.047555 -3.284475 3.040069

H -5.258018 -3.078794 0.106050

H -7.543847 1.922675 0.126593

H -6.698465 2.629886 -1.258098

H -7.209518 0.913855 -1.297354

H -2.665809 3.317987 2.126658

H -1.873727 1.772848 2.547365

H -3.587595 2.039710 2.959747

5S, 6S-C9 , Δ G = 1.0887 kcal/mol, population = 5.18 %

C -5.597430 1.227389 0.397085

C -4.994966 0.013284 0.466520

C -3.490133 0.121582 0.582504

C -3.178449 1.620165 0.456834

C -4.558352 2.280665 0.447754

C -5.599345 -1.362101 0.421697

C -6.189984 -1.238319 -2.088041

H -5.823290 -3.027919 -0.919843

H -4.377859 -2.070610 -1.225471

O -6.954123 -1.327386 0.836859

C -7.037304 1.613426 0.270607

O -4.760667 3.483723 0.477134

C -2.256628 2.205804 1.523782

H -3.165176 -0.281987 1.558165

H -2.982400 -0.489021 -0.182016

H -2.744643 1.824772 -0.538615

H -5.010421 -1.989810 1.121056

C -5.450418 -1.989053 -0.981124

C -6.101854 -1.942533 -3.440541

H -5.780301 -0.217435 -2.172247

H -7.245142 -1.120181 -1.791662

H -6.640654 -1.383172 -4.221592

H -6.536800 -2.954870 -3.393185

H -5.054052 -2.049059 -3.767840

H -7.315339 -2.219425 0.740524

H -7.702323 0.789828 0.546067

H -7.242294 2.495230 0.897864

H -7.260696 1.910909 -0.768846

H -2.183160 3.296975 1.402521

H -1.244847 1.777845 1.456378

H -2.648489 2.001064 2.533572

5S, 6S-C7 , Δ G = 1.3347 kcal/mol, population = 3.42 %

C -5.448130 1.249475 0.314891

C -4.757893 0.086317 0.233920

C -3.265248 0.285212 0.324778

C -3.069195 1.805518 0.454194

C -4.494334 2.366483 0.483421

C -5.335001 -1.294281 0.116089

C -6.173568 -1.466303 2.585087

H -4.289307 -2.211838 1.773445

H -5.721344 -3.076012 1.209633

O -4.560471 -1.983471 -0.858425

C -6.921212 1.497260 0.312514

O -4.786750 3.541463 0.632862

C -2.257411 2.260953 1.667378

H -2.862384 -0.259936 1.196430

H -2.770201 -0.146797 -0.558037

H -2.596335 2.207846 -0.458826

H -6.385554 -1.209697 -0.220809

C -5.334237 -2.070154 1.448710

C -5.443992 -0.443046 3.458164

H -6.520686 -2.289606 3.230712

H -7.089740 -1.016185 2.163561

H -6.091463 -0.092000 4.277226

H -5.127347 0.439621 2.885237

H -4.542465 -0.887007 3.911751

H -4.881809 -2.894797 -0.900316

H -7.285693 1.677099 1.338495

H -7.153099 2.403668 -0.267745

H -7.489469 0.652666 -0.101057

H -2.258616 3.359229 1.735461

H -1.214353 1.916320 1.599558

H -2.689032 1.860757 2.599448

5S, 6S-C10 , Δ G = 1.4132 kcal/mol, population = 2.99 %

C -5.365126 1.084434 0.145229

C -4.761022 -0.057200 0.558780

C -3.328212 0.165271 0.983643

C -3.045003 1.648120 0.695534

C -4.402591 2.202108 0.253610

C -5.319709 -1.452317 0.594285

C -5.486239 -3.626056 -0.767488

H -3.969874 -2.098080 -0.966887

H -5.568485 -1.598801 -1.542009

O -6.703654 -1.413487 0.907946

C -6.753098 1.334563 -0.352505

O -4.642022 3.377073 0.027424

C -2.437860 2.448241 1.846113

H -3.219781 -0.071779 2.057190

H -2.643168 -0.517006 0.452647

H -2.384008 1.736902 -0.185252

H -4.775009 -2.007730 1.384883

C -5.049022 -2.160636 -0.746756

C -5.193448 -4.314099 -2.099666

H -6.569582 -3.689527 -0.561527

H -4.981619 -4.167549 0.052521

H -5.521912 -5.365162 -2.090481

H -4.114214 -4.300478 -2.324763

H -5.711541 -3.807101 -2.930351

H -7.018954 -2.324650 0.974817

H -7.348261 0.416442 -0.373736

H -7.259745 2.074419 0.289845

H -6.718491 1.776821 -1.362169

H -2.373429 3.512506 1.573927

H -1.427192 2.089027 2.092975

H -3.061418 2.365311 2.751360

5S, 6S-C12 , Δ G = 1.5274 kcal/mol, population = 2.47 %

C -5.401686 1.171275 0.180610

C -4.824181 0.010048 0.577547

C -3.373654 0.185827 0.963762

C -3.048537 1.656792 0.660972

C -4.400151 2.256657 0.263785

C -5.425036 -1.365920 0.634885

C -5.723116 -3.532868 -0.743251

H -4.108201 -2.100512 -0.914688

H -5.669129 -1.499848 -1.498460

O -6.804481 -1.282792 0.958773

C -6.792709 1.470673 -0.280199

O -4.607991 3.439726 0.048717

C -2.371551 2.437219 1.785529

H -3.246166 -0.049355 2.035688

H -2.725963 -0.521756 0.419482

H -2.417354 1.718627 -0.243768

H -4.888915 -1.919777 1.430550

C -5.191016 -2.095598 -0.703703

C -5.064646 -4.482735 0.257883

H -5.578358 -3.920354 -1.765441

H -6.817626 -3.523368 -0.590631

H -5.435167 -5.511603 0.129360

H -5.267068 -4.192328 1.301482

H -3.970052 -4.500046 0.126306

H -7.151451 -2.182564 1.023431

H -7.414216 0.570524 -0.311633

H -7.265774 2.207618 0.390627

H -6.765792 1.937834 -1.278757

H -2.279667 3.497979 1.507762

H -1.365710 2.043017 1.996053

H -2.963466 2.378180 2.713608

5S, 6S-C8 , Δ G = 1.5895 kcal/mol, population = 2.22 %

C -5.387751 1.108263 0.192507

C -4.762748 -0.055958 0.495539

C -3.317815 0.143822 0.881737

C -3.044903 1.640467 0.663513

C -4.423471 2.221236 0.333583

C -5.351316 -1.443493 0.460985

C -4.189188 -2.028976 -1.773701

H -6.219590 -1.315748 -1.505837

H -5.943246 -2.969092 -0.929102

O -6.608207 -1.426601 1.125292

C -6.800662 1.369229 -0.221430

O -4.675797 3.408600 0.210549

C -2.361021 2.373894 1.814814

H -3.177087 -0.147847 1.938396

H -2.651242 -0.510759 0.296751

H -2.440561 1.774101 -0.252050

H -4.654491 -2.117982 0.997459

C -5.494239 -1.959850 -0.980796

C -4.373749 -2.642132 -3.160830

H -3.444891 -2.614879 -1.205512

H -3.768837 -1.013900 -1.876510

H -3.423185 -2.673641 -3.715970

H -5.093102 -2.059633 -3.759988

H -4.757819 -3.673438 -3.093232

H -7.017658 -2.294185 0.999079

H -7.444326 0.506992 -0.015629

H -7.189629 2.253301 0.308740

H -6.850840 1.604955 -1.298423

H -2.309663 3.451705 1.599276

H -1.337971 2.000827 1.974717

H -2.924686 2.240476 2.752640

5S, 6S-C13 , Δ G = 1.6598 kcal/mol, population = 1.97 %

C -5.509334 1.173966 0.293585

C -4.881991 -0.020421 0.446962

C -3.394823 0.133037 0.682371

C -3.115176 1.638163 0.558262

C -4.504044 2.257169 0.399674

C -5.451404 -1.409084 0.422476

C -5.139597 -1.573299 -2.169169

H -5.420498 -3.205332 -0.758640

H -3.860515 -2.407018 -0.636288

O -6.866453 -1.365252 0.480917

C -6.946668 1.521904 0.054546

O -4.736664 3.454793 0.373070

C -2.325382 2.267781 1.703911

H -3.135317 -0.249742 1.685258

H -2.809159 -0.467173 -0.032077

H -2.586108 1.840051 -0.390292

H -5.059262 -1.920882 1.326948

C -4.933927 -2.213217 -0.792205

C -6.596833 -1.447228 -2.617312

H -4.585925 -2.178339 -2.907439

H -4.663013 -0.577214 -2.179554

H -6.661575 -1.019068 -3.630443

H -7.173852 -0.810786 -1.933311

H -7.087946 -2.435009 -2.640709

H -7.195434 -2.266835 0.365852

H -7.625114 0.869741 0.614425

H -7.111670 2.573342 0.332616

H -7.202528 1.421579 -1.013839

H -2.267322 3.358396 1.569648

H -1.301653 1.866230 1.750335

H -2.815476 2.068957 2.671092

5S, 6S-C14 , Δ G = 2.0181 kcal/mol, population = 1.08 %

C -5.518665 1.262894 0.364152

C -4.882343 0.063674 0.408482

C -3.400282 0.204257 0.676855

C -3.130906 1.715989 0.680475

C -4.523635 2.338229 0.577913

C -5.464783 -1.314806 0.296146

C -5.179986 -1.698165 -2.265871

H -5.268021 -3.188658 -0.703242

H -3.775054 -2.262905 -0.695507

O -6.873791 -1.233420 0.183656

C -6.960901 1.611492 0.152003

O -4.762712 3.532261 0.661408

C -2.336072 2.252394 1.868796

H -3.152279 -0.257723 1.649098

H -2.800261 -0.330679 -0.075318

H -2.609719 1.998304 -0.252151

H -5.188449 -1.825048 1.244330

C -4.863921 -2.170810 -0.841995

C -4.360617 -0.503326 -2.757478

H -6.256379 -1.465770 -2.324225

H -5.015255 -2.547447 -2.950260

H -4.599139 -0.269742 -3.807332

H -3.279427 -0.712921 -2.700880

H -4.556619 0.402143 -2.165343

H -7.219603 -2.135646 0.149431

H -7.615970 1.092004 0.863570

H -7.083835 2.698276 0.268499

H -7.299658 1.327924 -0.856104

H -2.283559 3.350554 1.824895

H -1.310247 1.853550 1.875072

H -2.818291 1.971940 2.819525

5S, 6S-C11 , Δ G = 2.7134 kcal/mol, population = 0.33 %

C -5.698283 1.281620 0.710937

C -5.110440 0.084422 0.466984

C -3.620038 0.202523 0.258448

C -3.324618 1.710446 0.291429

C -4.664508 2.339365 0.684134

C -5.785672 -1.260466 0.376766

C -5.707438 -1.297343 -2.226761

H -7.349800 -0.659830 -0.964020

H -7.043609 -2.405394 -0.934950

O -6.667714 -1.407761 1.483593

C -7.129022 1.620641 0.982102

O -4.840848 3.520276 0.935003

C -2.183153 2.151168 1.205119

H -3.092205 -0.336863 1.065751

H -3.304135 -0.278673 -0.681276

H -3.116819 2.067949 -0.733276

H -4.999268 -2.037341 0.428391

C -6.548770 -1.416543 -0.952588

C -4.662210 -2.399811 -2.404852

H -5.220837 -0.306786 -2.254384

H -6.394522 -1.312618 -3.089123

H -4.136519 -2.294572 -3.366768

H -5.133172 -3.396726 -2.387479

H -3.898403 -2.384279 -1.611437

H -7.164889 -2.227774 1.353939

H -7.710001 0.728365 1.239185

H -7.191982 2.350668 1.804898

H -7.585330 2.104245 0.101192

H -2.123962 3.249635 1.233462

H -1.216663 1.758446 0.854387

H -2.345439 1.792041 2.234573

**Supplementary Data 2.** Cartesian Coordinates of stable conformers for diaportone C (**5**)

3S,4R,5R-C1 , Δ G = 0.0000 kcal/mol, population = 54.19 %

C -1.323259 0.093102 1.661768

C -1.592887 -1.097613 0.706401

C -0.269438 -1.261142 -0.047325

C 0.551034 0.017144 0.067785

C -0.235981 0.914908 0.976021

C 0.989866 -1.273252 0.754440

O -0.088179 2.114623 1.097878

C -2.715556 -0.764736 -0.270673

O -1.986781 -2.284633 1.377617

C 1.374562 0.587806 -1.075855

C 0.505168 1.409962 -2.034349

C 2.576801 1.387312 -0.559521

H -0.946588 -0.266407 2.634025

H -2.209107 0.709936 1.865456

H -0.290846 -1.801771 -0.997857

H 1.861198 -1.755588 0.305106

H 0.956675 -1.340065 1.845387

H -3.663745 -0.639913 0.273448

H -2.497866 0.166271 -0.814700

H -2.834717 -1.578845 -1.001305

H -1.231127 -2.602795 1.891807

H 1.762028 -0.281305 -1.636664

H 1.094009 1.751798 -2.900113

H -0.339414 0.812443 -2.412869

H 0.096051 2.298060 -1.529625

H 3.199229 1.742977 -1.396047

H 3.209888 0.769243 0.096769

H 2.238224 2.260122 0.017115

3S,4R,5R-C3 , Δ G = 0.4198 kcal/mol, population = 26.66 %

C -1.371710 0.170162 1.579360

C -1.546795 -1.094052 0.699166

C -0.184335 -1.253911 0.017087

C 0.585760 0.061585 0.085635

C -0.288273 0.984301 0.881813

C 1.030982 -1.164374 0.877514

O -0.201299 2.195632 0.893048

C -2.636428 -0.881922 -0.346438

O -1.917741 -2.248740 1.436209

C 1.458130 0.669672 -1.005402

C 2.694294 1.336684 -0.389219

C 1.845718 -0.329701 -2.097717

H -1.025743 -0.106639 2.589389

H -2.289990 0.760588 1.700304

H -0.142845 -1.871120 -0.883127

H 1.936244 -1.652656 0.509812

H 0.945174 -1.156530 1.967814

H -3.613338 -0.759802 0.145081

H -2.431038 0.016212 -0.947754

H -2.688363 -1.751052 -1.019346

H -1.174041 -2.489252 2.007156

H 0.849239 1.462264 -1.474298

H 3.290436 1.850190 -1.160324

H 2.399870 2.076913 0.368359

H 3.341124 0.586411 0.095040

H 2.474601 0.161932 -2.856127

H 0.961942 -0.739043 -2.609421

H 2.426174 -1.174271 -1.690708

3S,4R,5R-C2 , Δ G = 0.6156 kcal/mol, population = 19.15 %

C -1.226361 0.083626 1.697475

C -1.608755 -1.041667 0.701192

C -0.315366 -1.287407 -0.082631

C 0.610824 -0.088271 0.061395

C -0.078088 0.830511 1.029442

C 0.950292 -1.434179 0.696299

O 0.195477 2.001448 1.195727

C -2.713590 -0.579753 -0.243064

O -2.088804 -2.216786 1.335948

C 1.409083 0.555916 -1.064241

C 2.352838 -0.406355 -1.787070

C 0.455266 1.240200 -2.052962

H -0.875118 -0.346191 2.650452

H -2.052533 0.767443 1.935245

H -0.397927 -1.801025 -1.044647

H 1.759596 -1.992251 0.224714

H 0.924253 -1.527854 1.785668

H -3.639301 -0.391295 0.321169

H -2.422297 0.345710 -0.761326

H -2.913419 -1.355551 -0.997455

H -1.355603 -2.608589 1.831688

H 2.018962 1.341908 -0.585669

H 2.874265 0.116388 -2.603803

H 3.118781 -0.817621 -1.112981

H 1.802997 -1.250685 -2.236291

H 1.015580 1.786997 -2.827660

H -0.202468 1.958596 -1.541313

H -0.181276 0.494958 -2.557788
